# Discovery of Anomer-Inverting Transglycosylase: Cyclic Glucohexadecaose-Producing Enzyme from *Xanthomonas*, a Phytopathogen

**DOI:** 10.1021/jacs.4c02579

**Published:** 2024-06-19

**Authors:** Sei Motouchi, Shiro Komba, Hiroyuki Nakai, Masahiro Nakajima

**Affiliations:** † Department of Applied Biological Science, Faculty of Science and Technology, Tokyo University of Science, 2641 Yamazaki, Noda, Chiba 278-8510, Japan; ‡ Division of Food Processing and Biomaterials Biomaterials Development Group, Institute of Food Research, National Agriculture and Food Research Organization, 2-1-12, Kannondai, Tsukuba, Ibaraki 305-8642, Japan; § Faculty of Agriculture, Niigata University, 8050 Ikarashi 2-no-cho, Nishi-ku, Niigata 950-2181, Japan

## Abstract

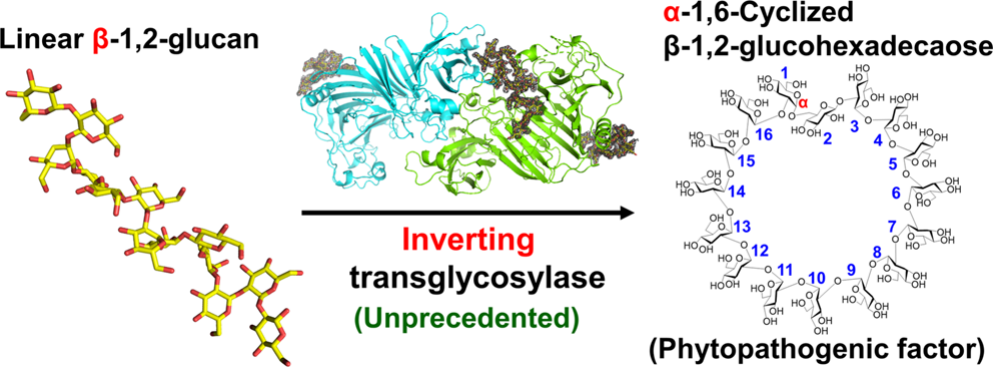

Various *Xanthomonas* species cause well-known plant diseases. Among various pathogenic factors, the role of α-1,6-cyclized β-1,2-glucohexadecaose (CβG16α) produced by *Xanthomonas campestris* pv. *campestris* was previously shown to be vital for infecting model organisms, *Arabidopsis thaliana* and *Nicotiana benthamiana*. However, enzymes responsible for biosynthesizing CβG16α are essentially unknown, which limits the generation of agrichemicals that inhibit CβG16α synthesis. In this study, we discovered that OpgD from *X. campestris* pv. *campestris* converts linear β-1,2-glucan to CβG16α. Structural and functional analyses revealed OpgD from *X. campestris* pv. *campestris* possesses an anomer-inverting transglycosylation mechanism, which is unprecedented among glycoside hydrolase family enzymes.

## Introduction

*Xanthomonas* species are generally plant pathogens that cause diseases in more than 400 different plant hosts such as rice, wheat, tomato, pepper, cabbage, cassava, banana, and bean.^1^ Although antimicrobial agrichemicals have been used mainly for avoiding virulence, antimicrobial-resistant bacteria that emerge by natural selection are a severe problem in agriculture. Conceptually new agrichemicals that inhibit pathogenicity without being invalidated by natural selection are in demand.

α-1,6-Cyclized β-1,2-glucohexadecaose (CβG16α) produced by *Xanthomonas campestris* pv. *campestris*(2) is a potential inhibition target for the agrichemicals described above.^3^ CβG16α is vital for the pathogenicity toward model plants, *Arabidopsis thaliana* and *Nicotiana benthamiana*, despite the nonproduction of CβG16α not affecting the fertility of *X. campestris* pv. *campestris.*(3) In detail, CβG16α suppresses the expression of pathogenesis-related (PR) proteins and callose accumulation in plants.^3^ However, key enzymes responsible for the biosynthesis of CβG16α remain unknown.

CβG16α is classified into osmo-regulated periplasmic glucans (OPGs).^4^ There are major patterns in the OPGs with a β-1,2-linked glucosyl backbone, namely, α-1,6-cyclized β-1,2-glucooligosaccharide, cyclic β-1,2-glucan (CβG), and β-1,2-glucooligosaccharide with β-1,6-glucose side chains (LβG-6β).^4^ Phenotypes of various mutants with genes related to the biosynthesis of mutated OPG (not limited to “OPG synthesis” itself) have been analyzed. Most of these mutants showed significantly different phenotypes from the wild-type species, such as lack of pathogenicity (*X. campestris* pv. *campestris*, *Brucella abortus*, *Agrobacterium tumefaciences*, *Pseudomonas syringae*, *Dickeya dadantii*, and *Salmonella enterica* sv. *typhimurium*) or symbiotic ability (some *Rhizobiaceae*).^4−11^ Although the enzyme synthesizing CβG has been identified, many other enzymes responsible for biosynthesizing the carbohydrate backbones of OPG have not been explored.^4^ For example, *Escherichia coli* synthesizes an LβG-6β-type OPG. However, even in *E. coli*, a well-known model organism, the biochemical functions of enzymes required after elongation of linear β-1,2-glucan were unknown.

Recently, Motouchi et al. identified a group of OPG-related proteins with unknown biochemical functions (MdoG superfamily) as a phylogenetically new glycoside hydrolase (GH) family, GH186. OpgD from *E. coli* (EcOpgD), a GH186-establishing enzyme, was elucidated to be a β-1,2-glucanase (SGL) (Note S1) that regulates the chain length of LβG-6β.^12^ A remarkable feature in GH186 is the amino acid sequence diversity of the loop A region, which is vital for sequestering the Grotthuss proton relay pathway for the reaction mechanism in EcOpgD. This observation indicates diverse functions and reaction mechanisms among GH186 enzymes, which are distributed mainly among α, β, and γ-proteobacteria, including *X. campestris* pv. *campestris.*(12)

**Figure 1:**
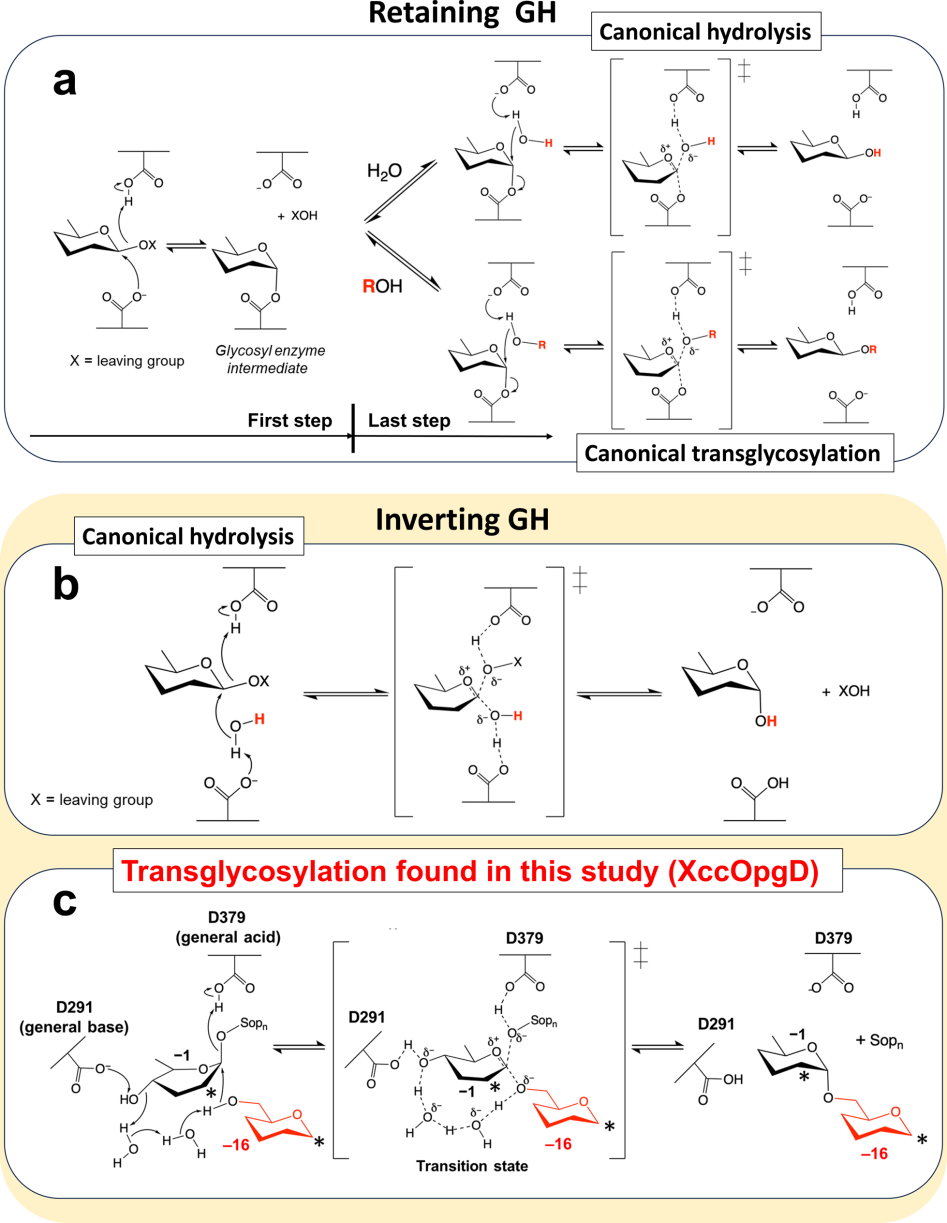
Proposed anomer-inverting transglycosylation mechanism of XccOpgD and comparison with canonical hydrolysis and transglycosylation. Arrows represent the pathways for electron transfer. The groups transferred to the anomeric position are shown as red letters or a red structural formula. (a–c) Reaction mechanism of anomer-retaining hydrolysis and transglycosylation (a), anomer-inverting hydrolysis (b), and transglycosylation (c). (c) Two asterisks at subsites −1 and −16 indicate the positions where a Sop_14_ unit is linked with β-1,2-glucosidic bonds. The n of Sop*_n_*s is likely to be 6 or higher according to the properties of XccOpgD.

In this report, we discovered that XccOpgD, a GH186 homologue from *X. campestris* pv. *campestris*, alone converts linear β-1,2-glucan into CβG16α specifically. Structural analysis of XccOpgD revealed an unprecedented reaction mechanism, anomer-inverting transglycosylation, providing the new concept of the reaction mechanisms among GH enzymes^13^ (Figure 1). Moreover, comparison of sequences between XccOpgD and the other GH186 homologues from bacteria including phytopathogens suggests further diversity of reaction products among GH186.

## Results

### Identification of the Reaction Product by XccOpgD

**Figure 2:**
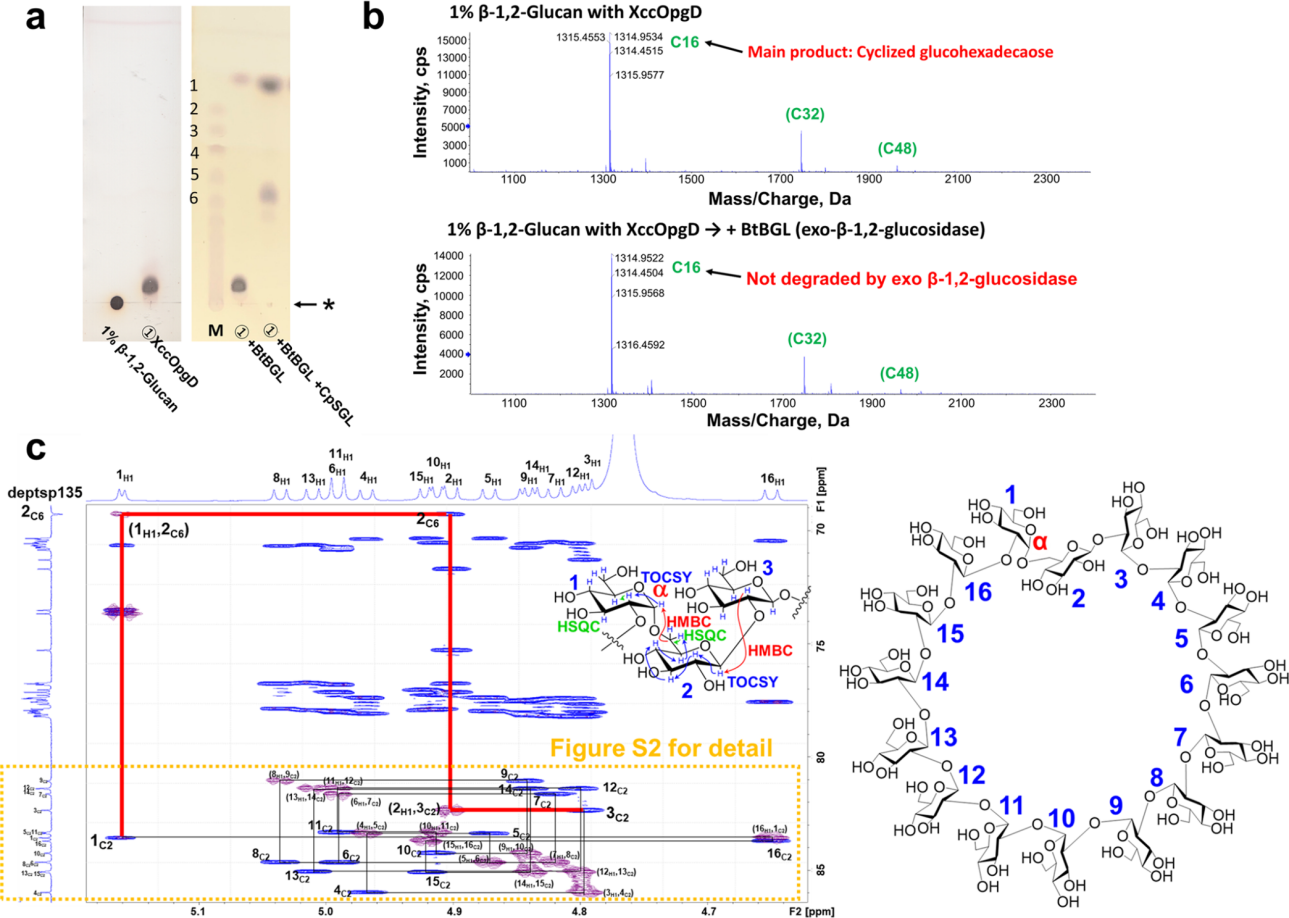
Identification of the main oligosaccharide produced by XccOpgD. (a), TLC analysis of the product produced by XccOpgD. Lane M, linear β-1,2-glucooligosaccharide marker prepared using 1,2-β-oligoglucan phosphorylase from *Listeria innocua*. DPs of linear β-1,2-glucooligosaccharides are shown on the left side of the TLC plates. The origins of the TLC plates are shown as horizontal arrows denoted by asterisks. ①, Linear β-1,2-glucan (average DP of 121, 1%) was incubated with XccOpgD (0.55 mg/mL) at 37 °C for 24 h. ① + BtBGL, BtBGL (the final concentration was 0.091 mg/mL) was added to ① and incubated at 37 °C for 24 h. ① + BtBGL + CpSGL, BtBGL and CpSGL (final concentrations were 0.083 and 0.25 mg/mL, respectively) were added to ① and incubated at 37 °C for 24 h. (b), Electrospray ionization-mass spectrometry analysis of the reaction products. The peaks are assigned as [M + *n*NH_4_]^*n*+^, and the arrow indicates the main product. Green letters and numbers represent forms of compounds (cyclic or linear) and DPs of products, respectively. For example, C16 represents cyclized hexadecaose. Green letters in parentheses are the peaks thought to be derived from cyclic glucohexadecaose. (top) Reaction products released from linear β-1,2-glucan by XccOpgD. (bottom) Reaction products after BtBGL treatment. The arrows next to the vertical axis represent the cutoffs that indicate the *m*/*z* value of the peak. (c), NMR analysis of the purified main product released by XccOpgD. Blue and purple peaks represent HSQC-TOCSY and HMBC data, respectively. The numbers of Glc moieties are defined by blue numbers. Black lines trace from 1_C2_ (2-carbon at the Glc moiety 1) to 3_C2_ (2-carbon at the Glc moiety 3) in the nonreducing end direction. Red lines trace from 3_C2_ to 1_C2_ in the nonreducing end direction.

Recombinant XccOpgD fused with a His_6_-tag at the C-terminus was produced by using *E. coli* as a host and purified successfully. XccOpgD exhibited activity toward linear β-1,2-glucans with an average degree of polymerization (DP) of 121 to produce glucans with adjusted DPs, and the amounts of products other than the main product were not at a detectable level by thin-layer chromatography (TLC) analysis (Figure 2a). The main product was not hydrolyzed by treatment with a β-glucosidase from *Bacteroides thetaiotaomicron* (BtBGL),^14^ an exotype enzyme (Figure 2a), indicating that the main product is cyclized or modified at the nonreducing end. It is considered that the thin spot of glucose detected after the treatment of BtBGL is derived from the undetectably smeared residual linear substrates (Figure 2a). In addition, the reaction seems to be highly biased to the product side (Note S2). Electrospray ionization-mass spectrometry (ESI-MS) analysis revealed that the DP of the product was 16, and the molecular mass was lower than that of linear β-1,2-glucan by 18 mass units, indicating that the product is a cyclic form (Figures 2b, S1 and Note S3). One- and two-dimensional NMR analyses were performed to determine the chemical structure of the product. Only one α-1,6-glucosidic bond was identified, with all other bonds being the β-1,2-glucoside type (Figure 2c; see Tables S1 and S2 and Supporting Information for further data). Thus, the product was identified as α-1,6-cyclized β-1,2-glucan consisting of 16 glucose units (α-1,6-cyclized β-1,2-glucohexadecaose, CβG16α) without side chains.

### Enzymatic Properties of XccOpgD

**Figure 3:**
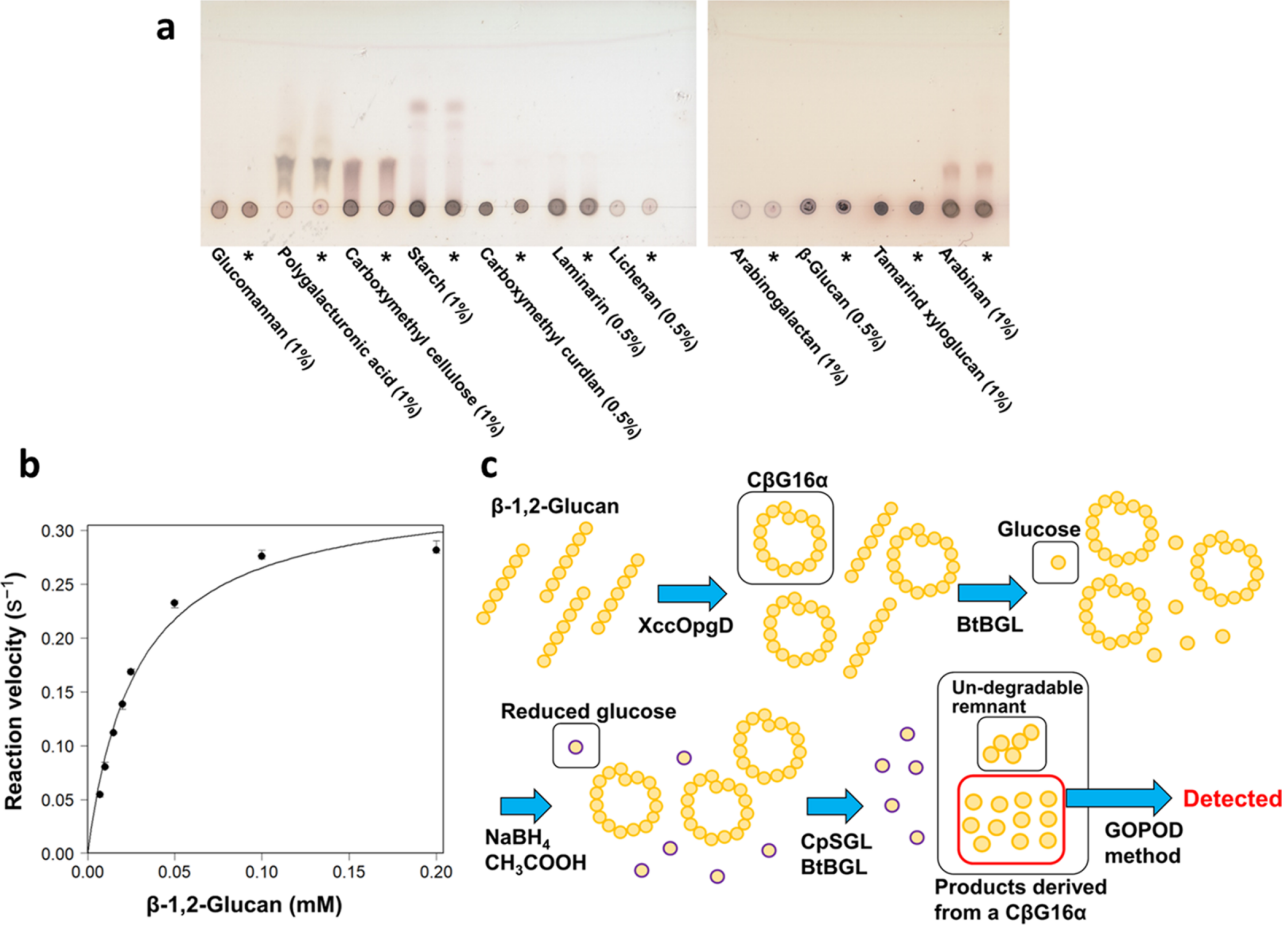
Enzymatic properties of XccOpgD. (a), Substrate specificity of XccOpgD. The reaction was performed at 37 °C for 24 h. Asterisks indicate that the reaction time was 24 h. The other lanes represent a reaction time of 0 h. (b), Kinetic analysis of XccOpgD. Data plotted as closed circles are medians from triplicate experiments, and the other data were used as error bars. Data were regressed with the Michaelis–Menten equation (solid line). (c), Strategy for quantifying the specific activity of XccOpgD.

XccOpgD exhibited the highest activity at 20–30 °C and pH 7.5 when linear β-1,2-glucans with an average DP of 121 were used as substrates (Figure S3a,b). XccOpgD exhibited no activity toward various polysaccharides, except for the linear β-1,2-glucan, indicating that XccOpgD is highly specific toward linear β-1,2-glucans (Figures 2a and 3a). Thus, kinetic analysis of XccOpgD was performed using linear β-1,2-glucan (average DP of 121) as the substrate (Figure 3b). A quantitative method for the cyclized product developed in this study is illustrated in Figure 3c (see the Methods for details). The *k*_cat_ value of XccOpgD was remarkably lower than that of SGLs (Table 1). The *K*_m_ value of XccOpgD was comparably lower than those of GH144 and GH162 SGLs. The result that both the *k*_cat_ and *K*_m_ values of XccOpgD were low is probably because the portion of the nonproductive complex between the substrate and the enzyme is high. In contrast, the *k*_cat_/*K*_m_ value of XccOpgD was similar to that of EcOpgD.

**Table 1:** Kinetic Parameters of XccOpgD for Linear β-1,2-Glucans and Comparison with Known SGLs

enzyme	*K*_m_ (mM)	*k*_cat_ (s^–1^)	*k*_cat_/*K*_m_ (s^–1^ mM^–1^)
XccOpgD	0.028 ± 0.0035	0.34 ± 0.015	12 ± 1.1
EcOpgD^a^ (GH186)	0.44 ± 0.056	29 ± 2	67 ± 3.5
CpSGL^a^ (GH144)	0.068 ± 0.014	49 ± 4	720 ± 110
TfSGL^a^ (GH162)	0.015 ± 0.001	31 ± 1	2100 ± 200

a The values for EcOpgD, CpSGL, and TfSGL are cited with permission from Motouchi et al.,^12^ Abe et al.,^15^ and Tanaka et al.,^16^ respectively.

### Michaelis Complex of XccOpgD

**Figure 4:**
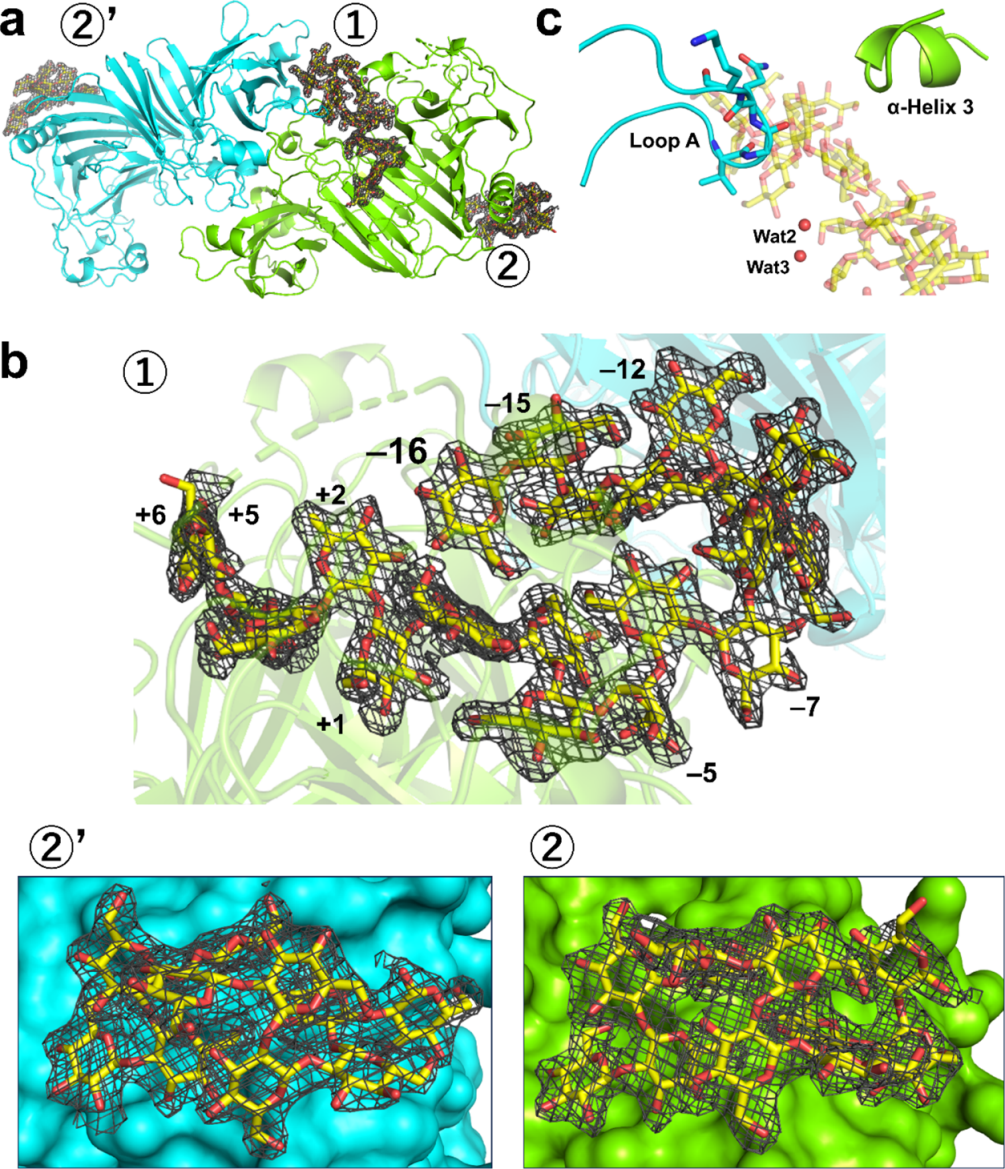
Structure of the Michaelis complex of XccOpgD. Chains A and B are colored light green and cyan, respectively. Linear β-1,2-glucans are shown as yellow sticks. (a), Asymmetric unit of XccOpgD and observed linear β-1,2-glucans. (b), Close-up view around each linear β-1,2-glucan. The electron densities of linear β-1,2-glucans are shown as the *F*_o_–*F*_c_ omit maps by gray meshes at the 3σ contour level. (c), Overview around Loop A and α-Helix 3. Wat2 and Wat3 are shown as red spheres. Linear β-1,2-glucan is shown semitranslucently.

The D379N mutant was used to determine a Michaelis complex structure of XccOpgD because D379 is conserved in GH186 and equivalent to the general acid of EcOpgD (Figure S4).^12^ The complex structure was obtained as a dimer (Figure 4a). Three glucan chains were observed in this structure. Two glucans have DPs of 11 and 10 and bind to sites distal from the catalytic pocket of XccOpgD (Figure 4a,b).

In the catalytic pockets of the dimer, the electron density of a linear β-1,2-glucan with DP22 was clearly observed only in Chain A (Figure 4b, top), probably because the closure motion of the cleft around α-Helix 3 is inhibited by crystal packing in Chain B (Figures 4c and S4), implying that the closure motion of the region is needed for substrate recognition. Superposition between Michaelis complexes of EcOpgD (PDB ID: 8IP1) and XccOpgD indicated that Chains A and B of the XccOpgD Michaelis complex form closed and open states, respectively (Figure S5). Thus, Chain A was used for describing the complex.

### Substrate Binding Mode of XccOpgD

**Figure 5:**
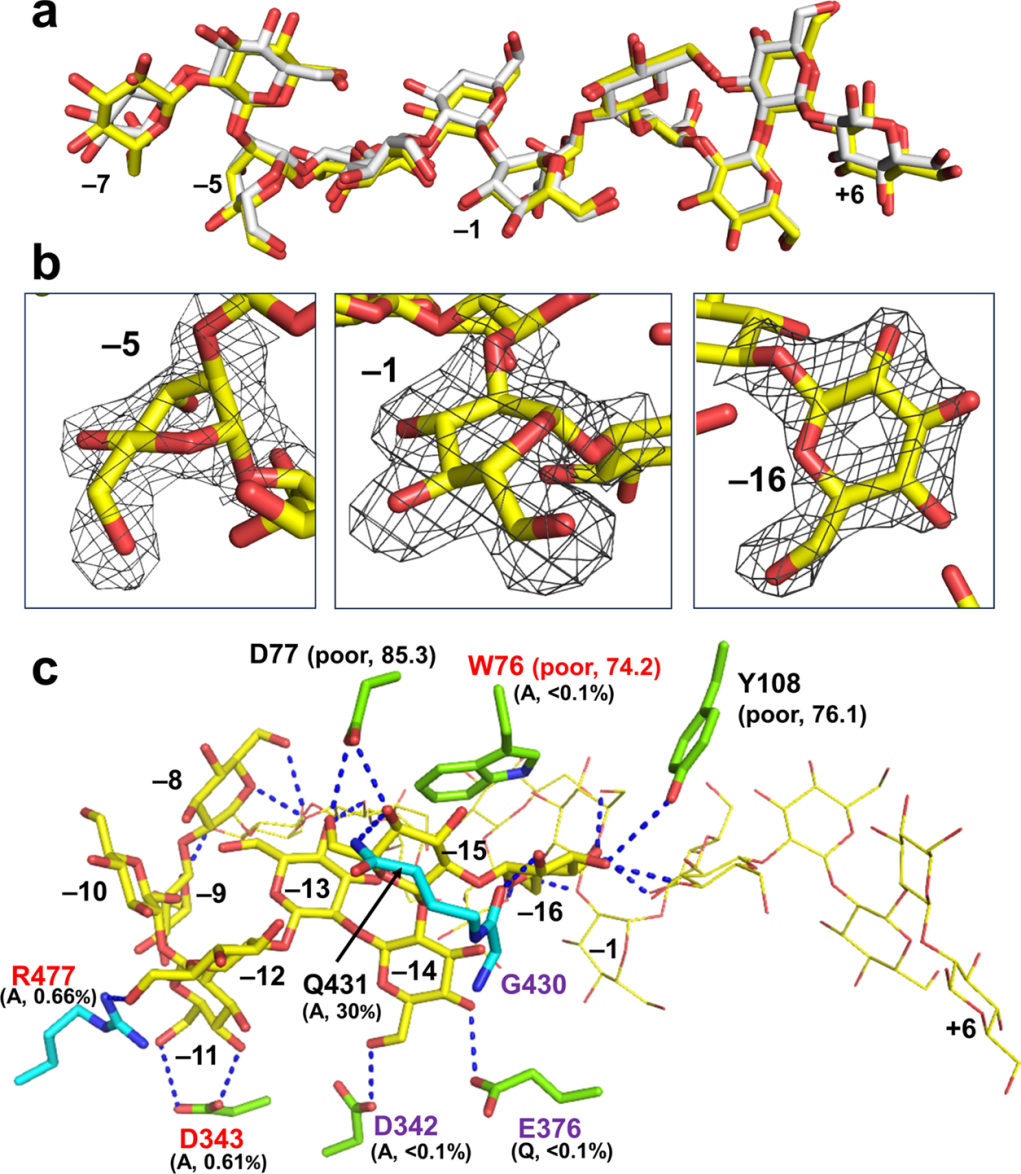
Substrate binding mode of XccOpgD. Linear β-1,2-glucans of XccOpgD and EcOpgD are shown as yellow and white sticks, respectively, except in part. (a), Superimposition of linear β-1,2-glucans of XccOpgD and EcOpgD at subsites −7 to +6. (b), Conformations of Glc moieties at subsites −5, −1, and −16 of XccOpgD. The electron density of each Glc moiety is shown as the *F*_o_–*F*_c_ omit map by a gray mesh at the 3σ contour level. (c), Substrate binding mode at subsites −16 to −8 of XccOpgD. The Glc moieties at subsites −7 to +6 are shown in a yellow line representation. Residues in Chains A and B are shown as light-green and cyan sticks, respectively. The relative activities (%) of mutants are shown below the substituted residues in parentheses with substituting residues. The residues highly conserved in GH186 are shown in purple letters. Residues whose mutations caused loss of cyclization activity despite not being conserved in GH186 are shown in red letters. Residues with relatively poor electron densities are indicated with average *B*-factors of the side chain atoms as (poor, *B*-factor).

Superimposed with the EcOpgD Michaelis complex, the positions and conformations of Glc moieties at subsites −7 to +6 (subsite is the nomenclature used for substrate binding sites; see https://www.cazypedia.org/index.php/Sub-site_nomenclature for details^17^) in XccOpgD are well conserved (Figure 5a). In particular, the distorted conformation (^1^S_3_) of the Glc moiety at subsite −1 is well superimposed (Figures 5b and S6). The subsite positions of XccOpgD are the same as EcOpgD after considering that such distortion generally causes energetic instability for the transition state in an enzymatic reaction (Figure 5a). In XccOpgD, the substrate is also observed from subsites −16 to −8 (Figure 5c). Subsite −16 is located near subsite −1, which appears to be suitable for cyclization (Figure 4b). This is consistent with the compound produced by XccOpgD being α-1,6-cyclized β-1,2-glucohexadecaose.

Glucose moieties at the subsite plus side are vital for the reaction as a leaving group thermodynamically. Consistently, electron densities at subsites +1 to +6 are obviously observed in the complex structure. Subsites +1 to +6 are likely to be important for substrate recognition because the electron densities at these plus subsites are clear even at a low substrate concentration as a preliminary result (data not shown, Note S4).

In EcOpgD, an α-helix moves drastically to form the catalytic cleft tunnel; however, P68 and N72 in α-Helix 3, which bind a substrate in the Michaelis complex, are not important for catalytic efficiency.^12^ In contrast, in XccOpgD, W76 in α-Helix 3 is likely to be important because this residue forms a stacking interaction with the Glc moiety at subsite −16 (Figures 4c, 5c and S6), and this is consistent with the W76A mutant displaying no cyclization activity (<0.1% specific activity compared with wild-type XccOpgD) (Table S3). In addition, the CD spectra of all mutants gave similar spectra (Figure S7). The Glc moiety at subsite −5 in EcOpgD is distorted (Figure 5b); however, the reason why such distortion is required is unclear.^12^ Interestingly, such distortion is conserved in XccOpgD, which is important for orientating a linear β-1,2-glucan to achieve a cyclic form (Figure 5).

There are relatively few substrate recognition residues at subsites −16 to −8 than at subsites −7 to +6 (Figures 5c and S4). Only intramolecular hydrogen bonds are found at four out of nine of these subsites (i.e., −8, −9, −10, and −13), which compensates for the stability of the binding mode (Figures 5c and S8). These properties of the binding mode might cause nonproductive binding such as binding only at subsites −7 to +6 (Table 1 and Figure 4). Among the substrate recognition residues for subsites −16 to −11, W76, D342, D343, E376, Q431, and R477 appear to be crucial (Figure 5c). W76A, D342A, D343A, E376Q, and R477A mutations severely disrupted the cyclization activity (Table S3 and Figure 5c). Considering fewer substrate binding residues, the effect of one mutation may be large. Among these mutated residues, D342 and E376 are highly conserved in GH186. The other residues are conserved only in limited clades, indicating that residues corresponding to W76, D343, and R477 may distinguish CβG16α synthases from other GH186 enzymes (see the Discussion Section for further detail).

### Transglycosylation Mechanism of XccOpgD

Anomer-inverting hydrolysis is the reaction in which the anomeric orientation at the scissile bond is inverted after hydrolysis. EcOpgD adopts an anomer-inverting hydrolytic mechanism, and the general acid and base are likely to be D388 and D300, respectively.^12^ A unique feature of the reaction mechanism is that D300 requires a route via the 4-hydroxy group of the Glc moiety at subsite −1 and two water molecules (Wat3 and Wat2) to deprotonate nucleophilic water (Wat1) by proton transfer (Figure S6).^12^

**Figure 6:**
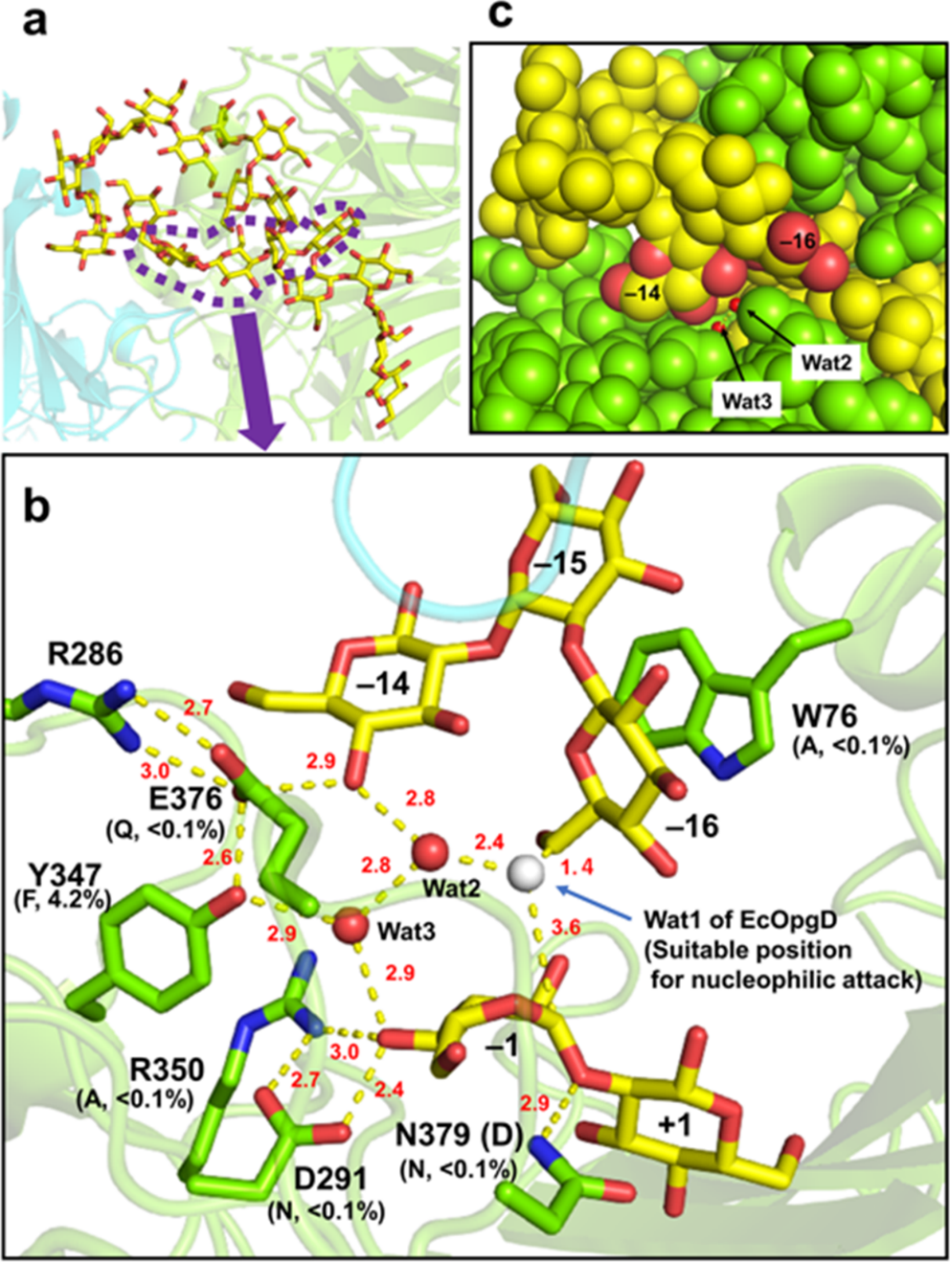
Environment around a linear β-1,2-glucan molecule bound to the catalytic cleft of XccOpgD. Hydrogen bonds are indicated by yellow dotted lines with lengths (red, Å). Residues and substrates are shown as green and yellow sticks, respectively. (a), Conformation of the whole linear β-1,2-glucan molecule in XccOpgD. Chains A and B are shown as semitransparent green and cyan cartoons, respectively. (b), Close-up view around subsites −14 and −16. The structure is superimposed with the Michaelis complex of EcOpgD (PDB ID: 8IP1) to show Wat1, a nucleophilic water (white sphere) of EcOpgD, as a suitable position for nucleophilic attack. The relative activities (%) of mutants are shown below the substituted residues in parentheses with substituting residues. N379 (D) represents the original residue in wild-type XccOpgD. (c), Sequestered proton transfer pathway of XccOpgD. Chain A and the substrate are shown as light-green and yellow spheres, respectively. Wat2, Wat3, and oxygen atoms of the Glc moieties at subsites −14 and −16 are shown as red spheres. Spheres except for Wat2 and Wat3 are shown with van der Waals radii.

To understand the reaction mechanism of XccOpgD, we compared XccOpgD with EcOpgD in terms of three key points: general acid, nucleophile, and general base. The general acid of EcOpgD (D388), located at a suitable position for protonating a glycosidic bond between subsites −1 and +1, is conserved in XccOpgD as D379. The Glc moiety at subsite −1 required for hydrolytic activity is distorted in XccOpgD, as observed for EcOpgD (Figures 6a,b and S6). Thus, D379 is probably the general acid, and the dramatic loss of cyclization activity (<0.1%) of the D379N mutant supports this residue acting as a general acid (Table S3).

Nevertheless, unlike EcOpgD, a nucleophilic water molecule is absent in XccOpgD. In XccOpgD, the 6-hydroxy group of the Glc moiety at subsite −16 is located near the anomeric carbon of the Glc moiety at subsite −1 (Figure 6b). Although the 6-hydroxy group does not adopt a suitable orientation for nucleophilic attack, the distance between Wat1 in EcOpgD and C6 of the Glc moiety at subsite −16 is 1.4 Å, which is a covalent bond distance (Figure 6b). This observation suggests that the 6-hydroxy group of the Glc moiety at subsite −16 can locate to a suitable position for nucleophilic attack (Note S5). Therefore, anomer-inverting transglycosylation will occur if the 6-hydroxy group is deprotonated.

Subsequently, to identify a general base, we traced the proton transfer pathway from Wat1 of EcOpgD, a potential position of the 6-hydroxy group of the Glc moiety at subsite −16. The pathway of Wat2-Wat3-O4 (subsite −1)-D291 of XccOpgD is the same as EcOpgD and the D291N mutant lost cyclization activity (<0.1%) (Figures 6b, S6 and Table S3), suggesting that D291 is a strong candidate for a general base. Nevertheless, E376 may also be a general base candidate (Figure 6b). The pathway of Wat2-Wat3-Y347-E376 is probably not the catalytic pathway because the Y347F mutant did not fully lose cyclization activity (4.2%) (Figure 6b and Table S3). The other pathway to reach E376, Wat2-O4 (subsite −14)-E376, may be possible because the E376Q mutant lost cyclization activity (<0.1%) (Figure 6b and Table S3). However, the remarkable decrease in cyclization activity for the E376Q mutant is probably because E376 is a substrate recognition residue at minus subsites near the nonreducing end, as described above. Indeed, D342A, which is the same mutant as E376Q in terms of losing one substrate recognition at subsite −14, showed a remarkably decreased cyclization activity (<0.1%) (Table S3 and Figure 5c). Therefore, we propose that D291 is the general base, whereas E376 is probably important for only substrate recognition.

## Discussion

α-1,6-Cyclized β-1,2-glucan is produced by well-known plant pathogens, such as *X. campestris* and *Ralstonia solanacearum*, and by model photosynthetic bacteria such as *Rhodobacter sphaeroides*.^2,4,18^ Interestingly, CβG16α produced by *X. campestris* was reported to be an immune-avoidance and infectious factor toward plants.^3^ However, no enzyme that synthesizes α-1,6-cyclized β-1,2-glucan has been identified. In this study, we focused on XccOpgD based on a perspective of functional diversity in the GH186 family and identified XccOpgD as the enzyme responsible for completing CβG16α synthesis. This enzyme should be given a new EC number. We propose linear (1 → 2)-β-d-glucan:(1 → 2)-β-d-glucohexadecaose 6-α-d-[(1 → 2)-β-d-glucohexadecaosyl]-transferase (cyclizing) as a systematic name and α-1,6-cyclized β-1,2-glucohexadecaose synthase as an accepted name.

Such cyclization of linear substrates arises from structural differences from EcOpgD, a hydrolase, in a small area. In EcOpgD, the long Grotthuss proton relay pathway from Wat1 was suggested to occur efficiently because Loop A (residues 434–453 a.a.) sequesters the pathway from the solvent^12^ (Figures S4 and S6). Although Loop A in XccOpgD is too short to cover the pathway for the Grotthuss proton relay, the pathway is sequestered from the solvent by the Glc moieties at subsites −14 and −16 (Figure 6c), which accounts for the efficient proton transfer of XccOpgD. Furthermore, the C6 atom in the glucose unit at subsite −16 is close to the position where nucleophilic attack to an anomeric carbon atom is possible. Hydrophobicity of the C6 atom may prevent a water molecule from accessing the appropriate position, which is the reason why transglycosylation specifically occurs in an environment full of water.^13^

Generally, it is needed for efficient transglycosylation by GH enzymes to avoid activation of a water molecule and/or exclude a water molecule from the position for nucleophilic attack.^13^ However, controlling the accessibility of a water molecule freely is still an open issue among anomer-retaining GHs containing various transglycosylases. In anomer-inverting GHs, even transglycosylation itself had not been found until this study. Functional and structural analyses of XccOpgD in this study evidenced that appropriate assignment of a hydroxy group in a glycan for nucleophilic attack and appropriate sequestration of a proton relay pathway can achieve transglycosylation, even in anomer-inverting GHs. Thus, it can be said that anomer-inverting transglycosylation proposed in Figure 1 is the first extremely special case among GH family enzymes (Note 6).

This study corroborated the diversity of the reaction mechanisms in the GH186 family and provided perspectives on functionally unknown clades in GH186. Mutational analyses revealed that W76, D343, and R477 are important substrate recognition residues for the cyclization activity of XccOpgD to produce CβG16α. These residues represent indicators for distinguishing α-1,6-cyclized β-1,2-glucohexadecaose synthases from other GH186 homologues. In GH186, these three residues are highly conserved in only clade 3, which is next to the clade of EcOpgD in the phylogenetic tree (Figure S9a). Although W76 of XccOpgD is substituted with Tyr in some clade 3 homologues, the stacking interactions with the Glc moiety at subsite −16 are expected to be conserved, indicating that clade 3 homologues share efficient cyclization activity (Figure S9c).

In clade 4, W76 of XccOpgD is substituted with Tyr (Figure S9d), as observed for some homologues in clade 3, implying that homologues in clade 4 retain anomer-inverting transglycosylation activity. However, D343 and R477, important residues in XccOpgD, are not conserved in clade 4 (Figure S9d), indicating that these homologues do not synthesize CβG16α.

In clade 2, particular homologues near clade 3 have conserved residues corresponding to R477 and D343 of XccOpgD, implying that they recognize the Glc moieties at minus subsites, which are important for locating linear β-1,2-glucan as a suitable form for cyclization (Figure S9b). Nevertheless, these homologues have a highly conserved GW motif on Loop A, which is vital for stabilizing the nucleophilic water required for hydrolytic activity.^12^ Furthermore, W76 of XccOpgD is a proline, which cannot form stacking interactions with a Glc moiety, suggesting that these homologues are not transglycosylases but hydrolases. Thus, it is likely that the homologues described above are intermediates of EcOpgD and XccOpgD and that the significant changes in the reaction mechanisms arose from the minor sequence variations described above.

Overall, GH186 homologues that produce α-1,6-cyclized β-1,2-glucohexadecaose are hypothesized to be limited to clade 3, and the function of many GH186 homologues differs from XccOpgD and EcOpgD. Homologues of most *Xanthomonas* pathogens are located specifically in clade 3, implying that many *Xanthomonas* diseases can be regulated by inhibiting α-1,6-cyclized β-1,2-glucohexadecaose synthase. Furthermore, given that the environment for capturing subsites −7 to +6 is highly conserved in the majority of GH186 enzymes, GH186 has the potential to be a broadly adaptable inhibition target for treating *Xanthomonas* diseases related to CβG16α and for other animal or plant pathogens that use other OPGs such as LβG-6β.

## Supporting Information Available

Electrospray ionization-mass spectrometry analysis; NMR analysis of the β-1,2-glucosidic bonds of the main product produced by XccOpgD; H-1, H-2 and H-6 proton chemical shifts of the product produced by XccOpgD; specific activities of XccOpgD mutants; NMR data for CβG16α (PDF)

## Abbreviations

CβG16α: α-1,6-cyclized β-1,2-glucohexadecaose
OPG: osmo-regulated periplasmic glucans
CβG: cyclic β-1,2-glucan
LβG-6β: β-1,2-glucooligosaccharide with β-1,6-glucose side chains
GH: glycoside hydrolase
EcOpgD: OpgD from *E. coli*
SGL: β-1,2-glucanase
XccOpgD: OpgD from *X. campestris* pv *campestris*
DPs: degrees of polymerization
TLC: thin-layer chromatography
BtBGL: β-glucosidase from *B. thetaiotaomicron*
ESI-MS: electrospray ionization-mass spectrometry
Glc: glucose
