# Supplementary material for: Discovery of Anomer-Inverting Transglycosylase: Cyclic Glucohexadecaose-Producing Enzyme from Xanthomonas, a Phytopathogen
Source: J Am Chem Soc. 2024 Jun 19;146(26):17738–46. doi: 10.1021/jacs.4c02579 (PMC11228985; doi:10.1021/jacs.4c02579)
Supplement: Supplementary file 1 — ja4c02579_si_001.pdf [file ja4c02579_si_001.pdf]

Supplementary information

Discovery of anomer-inverting transglycosylase: cyclic glucohexadecaose-producing enzyme from *Xanthomonas*, a phytopathogen.

Sei Motouchi[1]*, Shiro Komba[2], Hiroyuki Nakai[3] & Masahiro Nakajima[1]*

[1]Department of Applied Biological Science, Faculty of Science and Technology, Tokyo University of Science, 2641 Yamazaki, Noda Chiba 278-8510, Japan.

[2] Division of Food Processing and Biomaterials Biomaterials Development Group, Institute of Food Research, National Agriculture and Food Research Organization, 2-1-12, Kannondai, Tsukuba, Ibaraki 305-8642, Japan.

[3]Faculty of Agriculture, Niigata University, 8050 Ikarashi 2-no-cho, Nishi-ku, Niigata 950-2181, Japan.

*Masahiro Nakajima
E-mail: m-nakajima@rs.tus.ac.jp

*Sei Motouchi
E-mail: 6423703@ed.tus.ac.jp

Table of Contents

Supplementary Figures S1-S9
Supplementary Tables S1-S5
Supplementary Notes S1-S6
Experimental Methods
Supporting References

Supplementary Figures

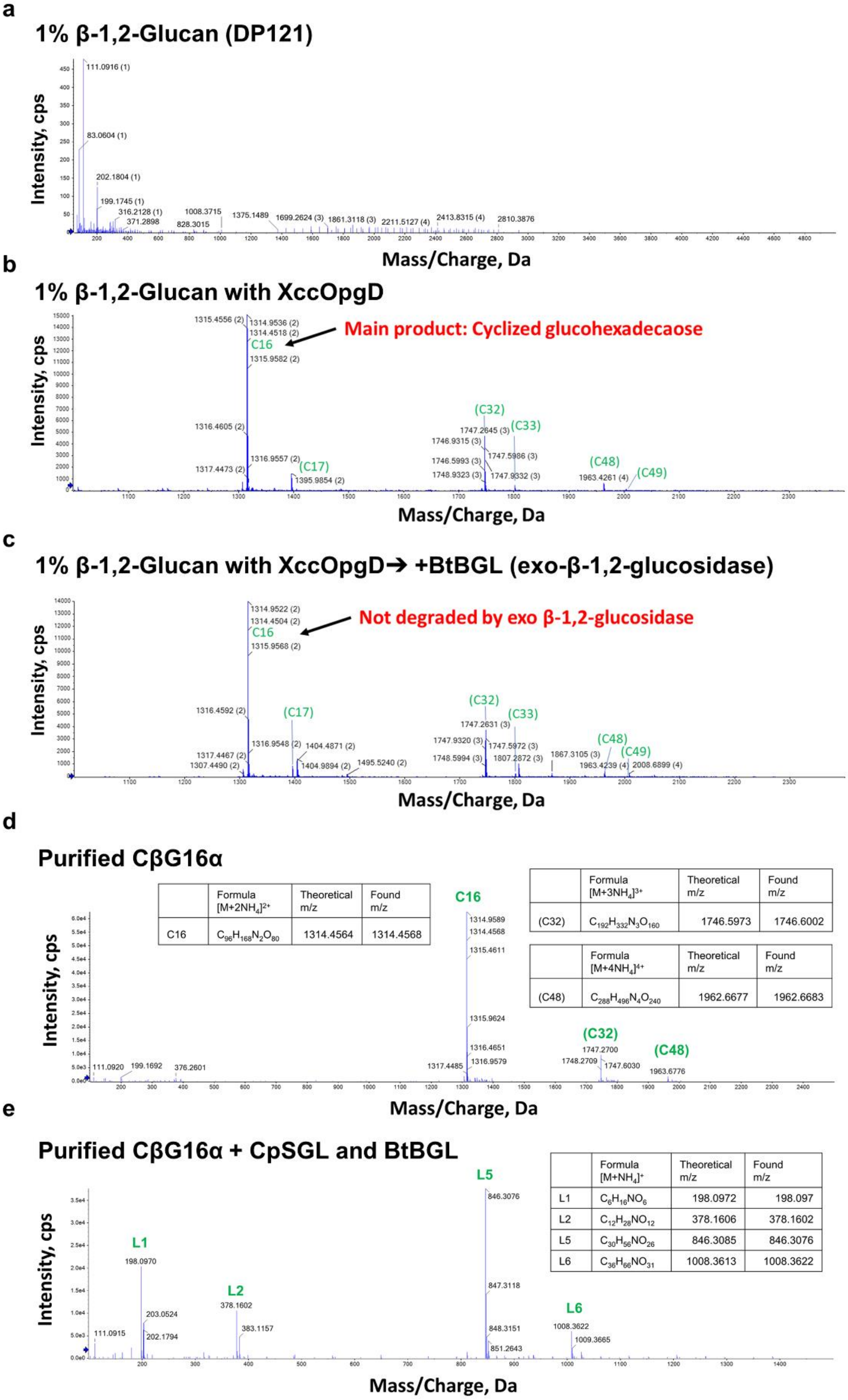

	Formula $[M+2NH_4]^{2+}$	Theoretical m/z	Found m/z
C16	$C_{96}H_{168}N_2O_{80}$	1314.4564	1314.4568

	Formula $[M+3NH_4]^{3+}$	Theoretical m/z	Found m/z
(C32)	$C_{192}H_{332}N_3O_{160}$	1746.5973	1746.6002

	Formula $[M+4NH_4]^{4+}$	Theoretical m/z	Found m/z
(C48)	$C_{288}H_{496}N_4O_{240}$	1962.6677	1962.6683

	Formula $[M+NH_4]^{+}$	Theoretical m/z	Found m/z
L1	$C_6H_{16}NO_6$	198.0972	198.097
L2	$C_{12}H_{28}NO_{12}$	378.1606	378.1602
L5	$C_{30}H_{56}NO_{26}$	846.3085	846.3076
L6	$C_{36}H_{66}NO_{31}$	1008.3613	1008.3622

Figure S1. Electrospray ionization-mass spectrometry analysis. The peaks are assigned as $[M + nNH_4]^{n+}$ and indicated by arrows for the products. Green text represents forms of compounds (cyclic or linear) and degrees of polymerization (DPs) of products. For example, L5 represents a linear pentaose. The arrows next to the vertical axis represent the cutoffs which indicate the m/z value of the peak. **a–e**, ESI-MS data of linear β-1,2-glucan (**a**), the reaction products released from linear β-1,2-glucan by XccOpgD (**b**), the reaction products after BtBGL treatment (**c**), purified CβG16α (**d**) and the reaction products released from CβG16α following treatment with BtBGL and CpSGL (**e**).

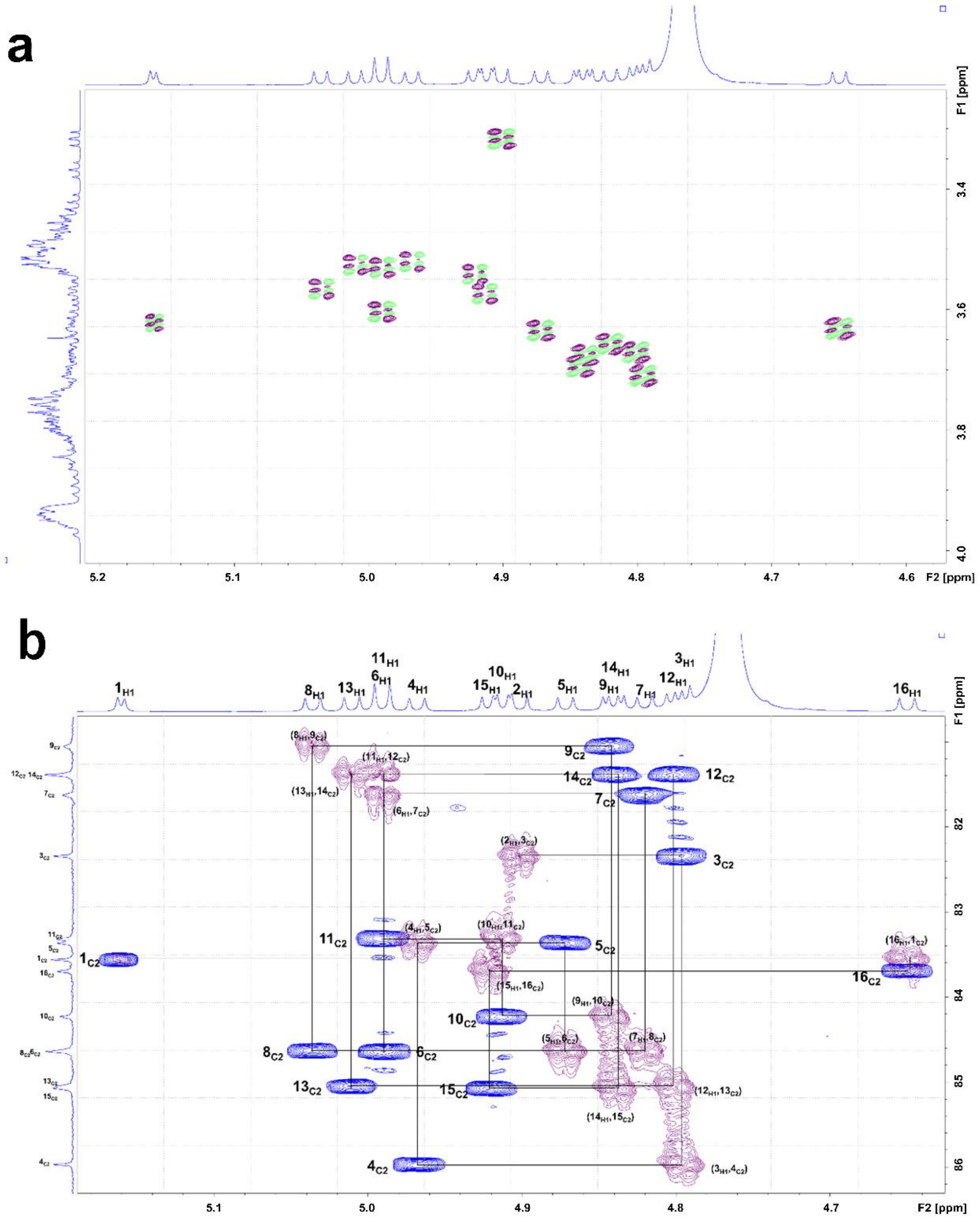

Figure S2. NMR analysis of the β-1,2-glucosidic bonds of the main product produced by XccOpgD. a, The 2D ^{1}H-^{1}H COSY spectrum of the main product from XccOpgD. Green and brown peaks represent COSY correlations between H1 and H2. **b**, Overlay of 2D HSQC-TOCSY and HMBC spectra of the main product from XccOpgD. Blue and purple peaks represent HSQC-TOCSY and HMBC correlations, respectively. The number labels beside the peaks are the numbers assigned for Glc moieties in Fig. 1c. Black lines trace from the 1_{C2} (2-carbon at the Glc moiety 1) to 2_{H1} (1-proton at the Glc moiety 2) in the direction to the non-reducing end.

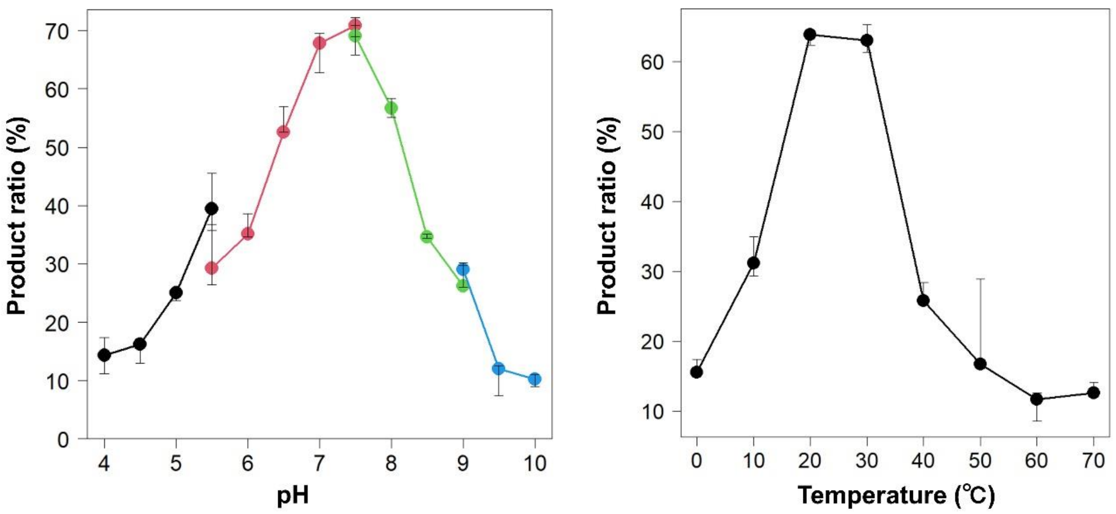

Figure S3. pH and temperature profiles of XccOpgD. a, pH optimum. Buffers used for the enzymatic reactions were sodium acetate-HCl (pH 4.0–5.5, black), bis-Tris-HCl (pH 5.5–7.5, red), Tris-HCl (pH 7.5–9.0, green) and glycine-NaOH (pH 9.0–10, blue). b, Temperature optimum. Data plotted as closed circles are medians from triplicate experiments, and the other data were used for error bars (a, b).

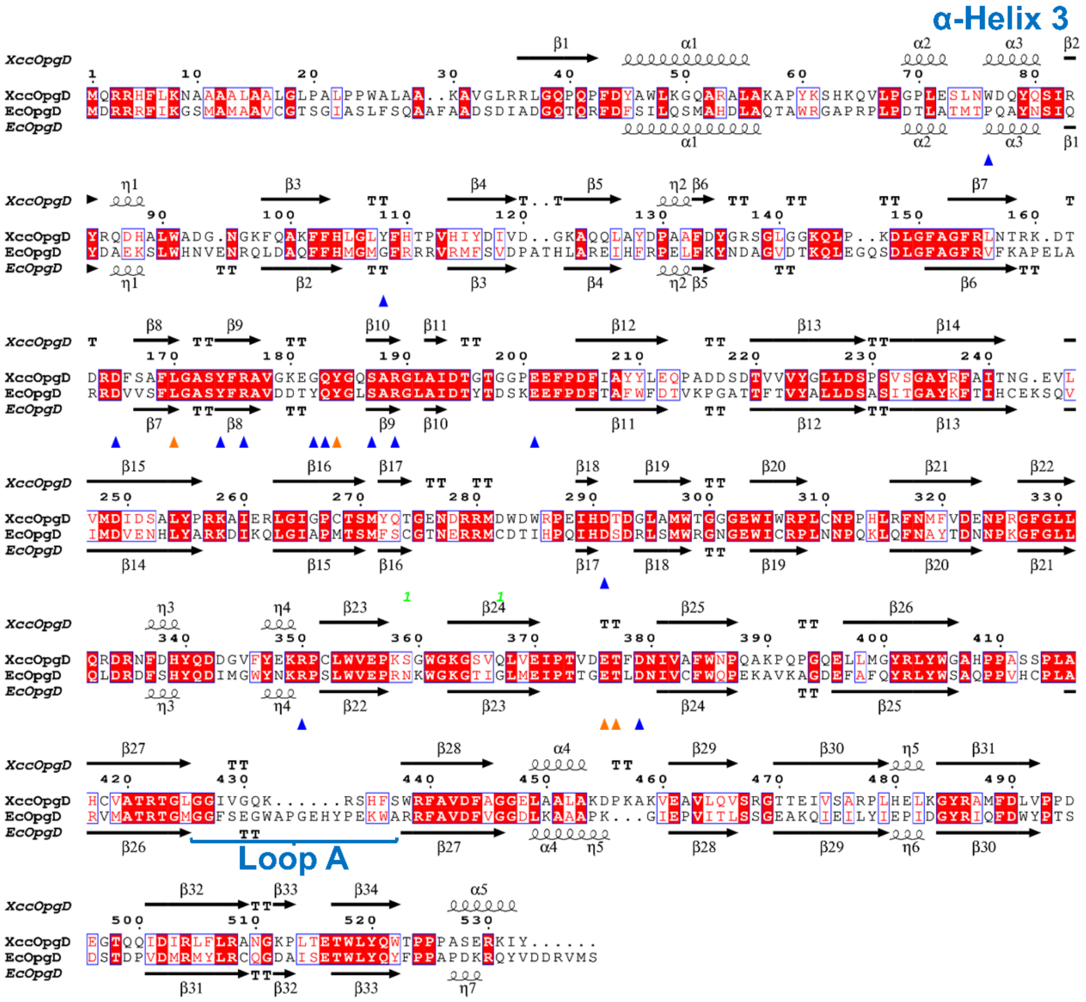

Figure S4. Pairwise sequence alignment of XccOpgD and EcOpgD. The alignment was performed using Clustal Omega and visualized using the ESPript 3.0 server (http://espript.ibcp.fr/ESPript/ESPript/). The secondary structures of XccOpgD and EcOpgD are shown above and below the sequences. The regions of α-Helix 3 and Loop A are highlighted in blue letters. Blue and orange triangles represent substrate recognition residues via side chains and only main chains at subsites −7 to +6 of XccOpgD, respectively.

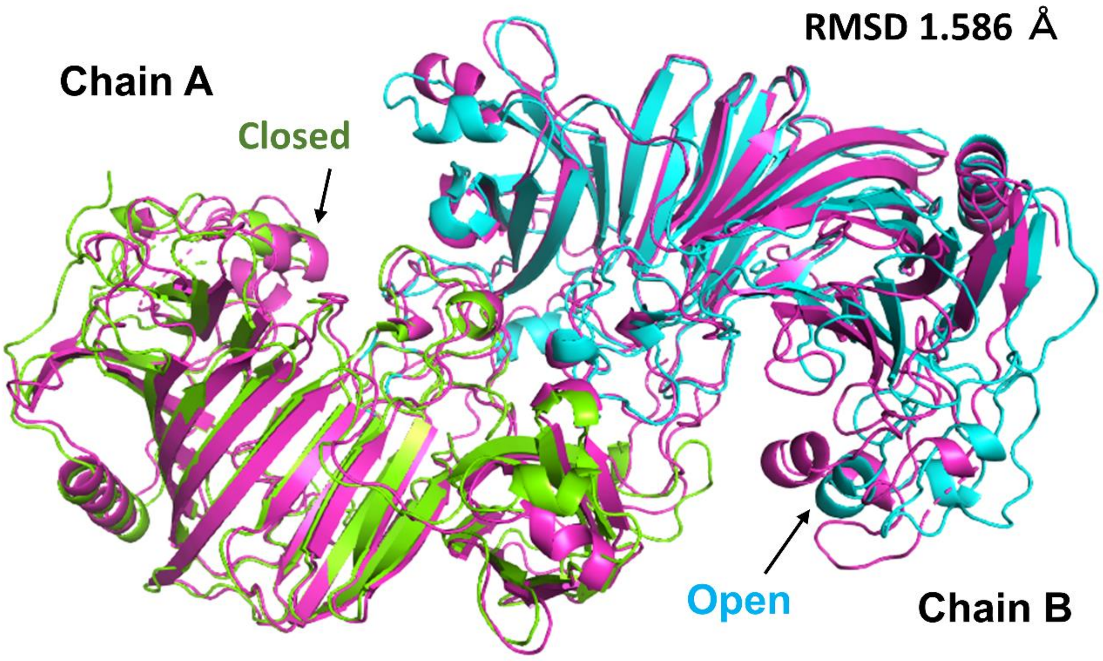

Figure S5. Superposition between Michaelis complexes of XccOpgD and EcOpgD. EcOpgD (PDB ID: 8IP1) is shown as a purple cartoon. Chains A and B of XccOpgD are shown as light green and cyan cartoons, respectively. Substrates are omitted. Chains A and B of EcOpgD form a closed state. In contrast, chains A and B of XccOpgD form closed and open states, respectively, because of crystal packing effects. The root mean square of deviation (RMSD) between XcOpgD and EcOpgD is 1.586 □.

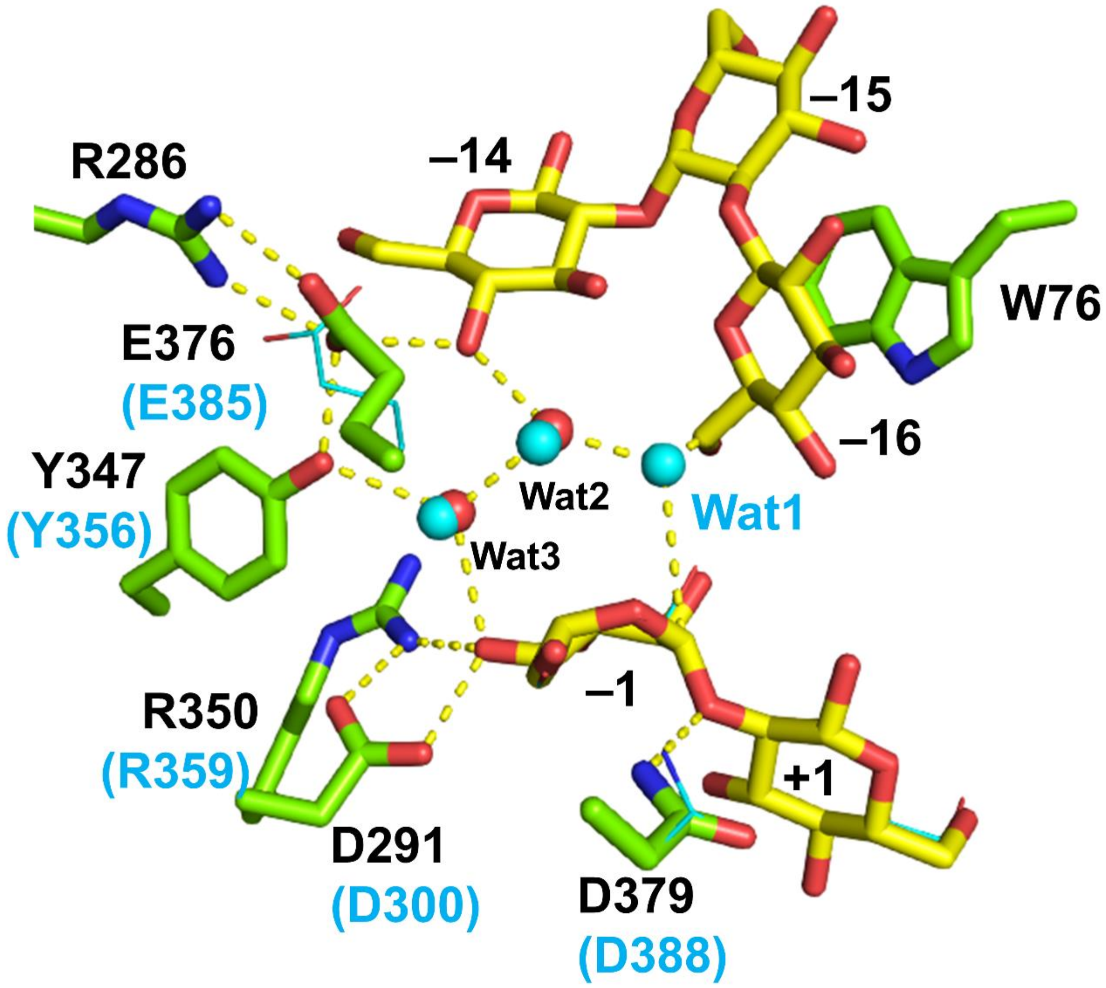

Figure S6. Superimposition between reaction centres of XccOpgD and EcOpgD. Chain A and the substrate of the XccOpgD complex structure (PDB ID: 8X18) are shown as light green and yellow sticks, respectively. Black and cyan labels represent residues in XccOpgD and EcOpgD, respectively. Chain A and water molecules of the superimposed EcOpgD complex structure (PDB ID: 8IP1) are shown as cyan lines and spheres, respectively.

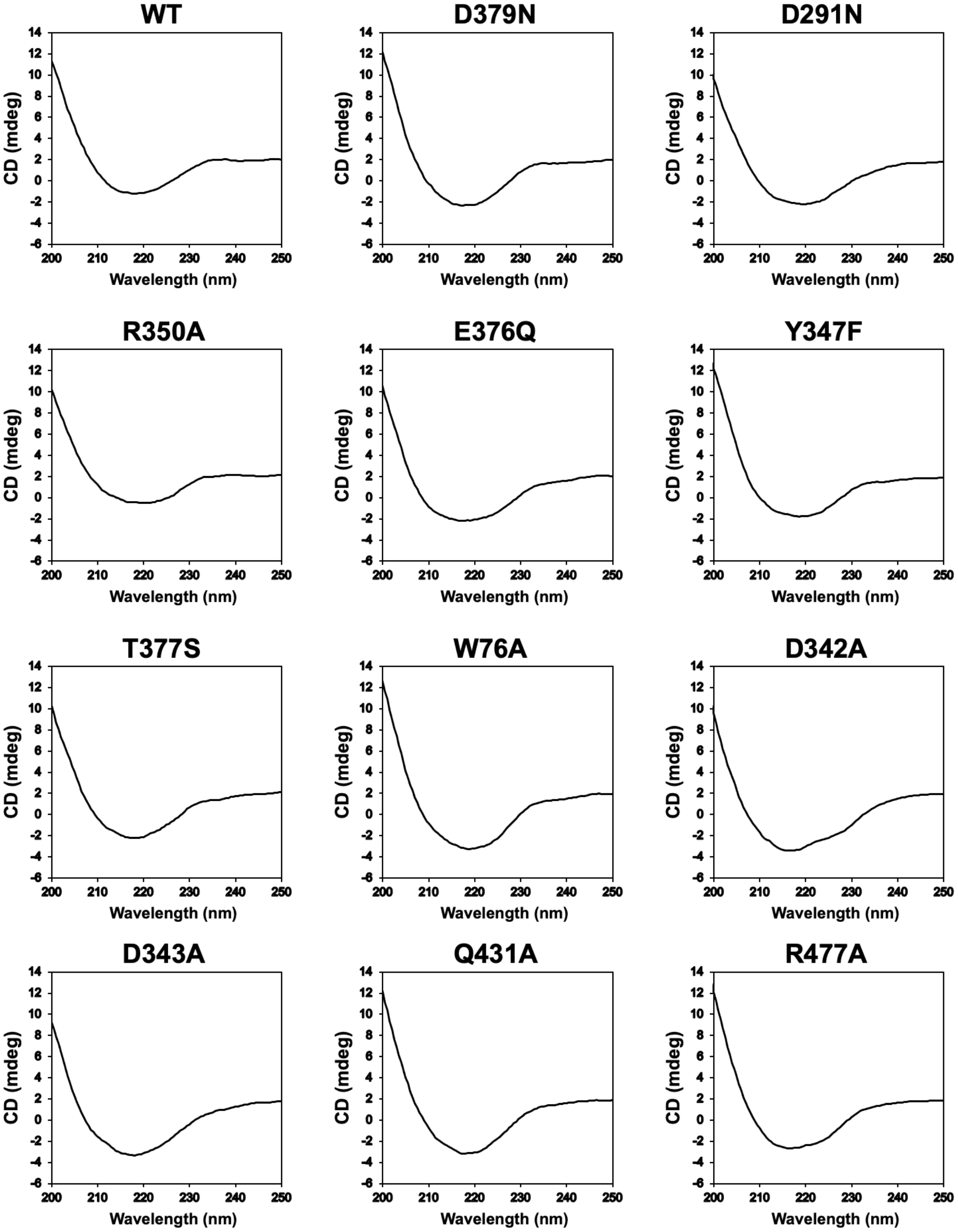

Figure S7. CD spectra of wild-type (WT) XccOpgD and mutants.

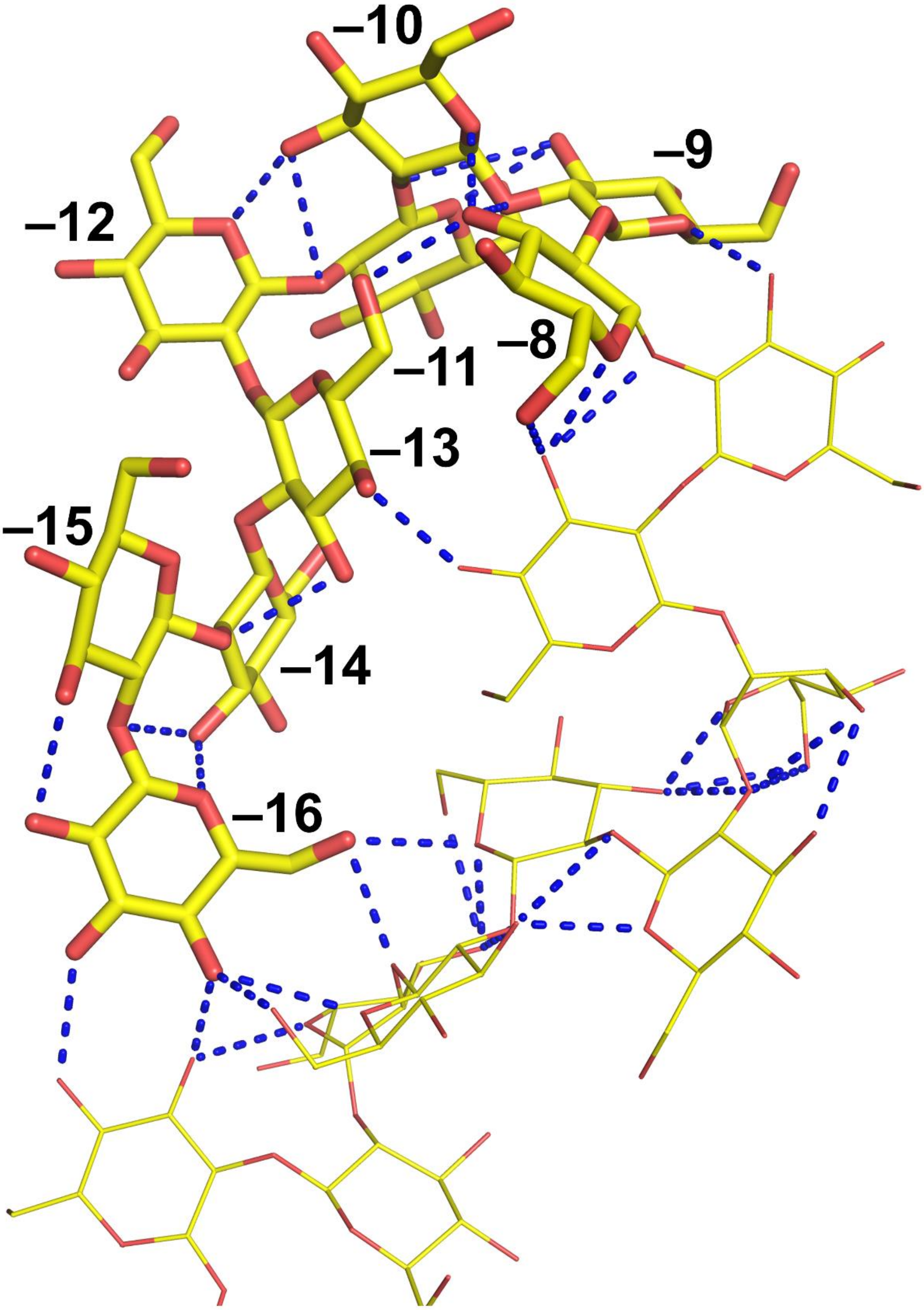

Figure S8. Intramolecular interactions of the β-1,2-glucan at subsites −16 to −8. Substrates of subsites −16 to −8 and −7 to +6 are shown as yellow sticks and lines, respectively. Intramolecular hydrogen bonds are shown as dashed lines. Subsite positions are labelled with numbers.

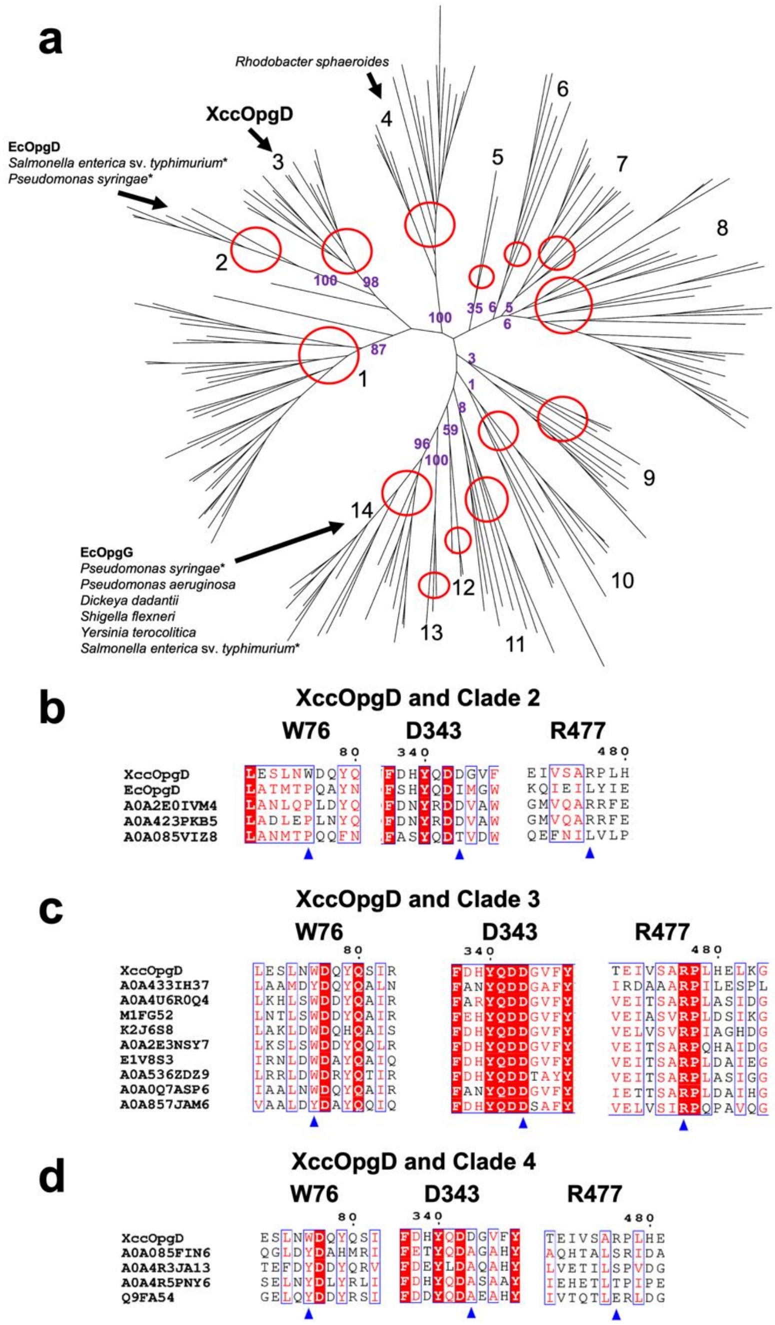

Figure S9. Phylogenetic tree of GH186 and multiple sequence alignments. a, Each clade is indicated by a red circle with a clade number. Purple letters indicates the bootstrap values. Black arrows indicate clades, including homologues from Gram-negative bacteria whose phenotypes of OPG-related gene knockout mutants have been investigated. Asterisks represent species possessing two homologues. **b–d**, Multiple sequence alignments of clades 2–4. The alignments were performed using Clustal Omega (https://www.ebi.ac.uk/Tools/msa/clustalo/) and are visualized using the ESPript 3.0 server (http://espript.ibcp.fr/ESPript/ESPript/). The homologues are represented by UniProt accession numbers. Residue numbers of XccOpgD are shown above the alignments.

Supplementary Tables

Table S1. H-1, H-2 and H-6 proton chemical shifts of the product produced by XccOpgD

800 MHz NMR (D_2O with internal standard tBuOH δ = 1.23, 298 K)

Proton	Chemical shift of each glucose residue (ppm)															
	1	2	3	4	5	6	7	8	9	10	11	12	13	14	15	16
H-1 (ppm)	5.160	4.902	4.796	4.968	4.872	4.991	4.820	5.036	4.843	4.914	4.991	4.801	5.010	4.839	4.921	4.650
$J_{1,2}$ (Hz)	3.48	7.99	7.78	7.93	7.90	7.91	7.87	7.88	7.89	7.87	7.91	7.92	7.93	7.90	7.86	7.91
H-2[a] (ppm)	3.62	3.32	3.71	3.52	3.63	3.53	3.66	3.56	3.69	3.57	3.60	3.67	3.53	3.68	3.54	3.63
H-6[b] (ppm)	A[c]	3.85	A[c]	A[c]	A[c]	A[c]	A[c]	A[c]	A[c]	A[c]	A[c]	A[c]	A[c]	A[c]	A[c]	A[c]

[a]H-2 chemical shifts were determined using ^{1}H-^{1}H COSY NMR data.

[b]H-6 chemical shifts were determined using DEPT135, HSQC, HSQC-TOCSY and HMBC NMR data.

[c]A: 3.73–3.98 ppm.

Table S2. C-1, C-2 and C-6 carbon chemical shifts of the product produced by XccOpgD

800 MHz NMR (D_2O with internal standard tBuOH δ = 31.30, 298 K)

Carbon	Chemical shift of each glucose residue (ppm)															
	1	2	3	4	5	6	7	8	9	10	11	12	13	14	15	16
C-1 (ppm)	100.02	104.03	104.73	103.28	103.94	102.85	103.65	102.80	103.91	103.46	102.69	104.16	102.98	104.18	103.94	104.80
C-2 (ppm)	83.55	75.26	82.33	85.96	83.36	84.63	81.62	84.63	81.05	84.22	83.31	81.38	85.04	81.38	85.06	83.69
C-6 (ppm)	A[a]	69.26	A[a]	A[a]	A[a]	A[a]	A[a]	A[a]	A[a]	A[a]	A[a]	A[a]	A[a]	A[a]	A[a]	A[a]

[a]A: 62.16–62.84 ppm.

Table S3. Specific activities of XccOpgD mutants

	Specific activity (U/mg)	Relative activity (%)
D379N	ND	ND
D291N	ND	ND
R350A	ND	ND
E376Q	ND	ND
Y347F	0.012 (0.00081)	4.2
T377S	0.016 (0.00030)	5.5
W76A	ND	ND
Q431A	0.086 (0.0059)	30
R477A	0.0019 (0.00025)	0.66
D342A	ND	ND
D343A	0.0018 (9.8×10^{-5})	0.61
Wild-type	0.29	100

All specific activities were measured using 0.1 mM linear β-1,2-glucan as the substrate. Medians of triplicate experiments are presented. ND represents data of less than 0.00029 units/mg (0.1% relative activity). Maximum absolute values between medians and the other data of the triplicate experiments are shown in parentheses. Activity to produce 1 μmol of reaction product per minute is defined as 1 U (μmol/min).

Table S4. Primer pairs for XccOpgD mutants

	Forward primer	Reverse primer
D379N	accttcaacaacatcgtggcgttctgg	gatgttgttgaaggtctcgtccacggt
D291N	atccacaataccgatggcctggcgatg	atcggtattgtggatttctgggcgcca
R350A	gagaaggctccgtgcctgtgggtggag	gcacggagccttctcgtagaacacgcc
E376Q	gtggaccaaaccttcgacaacatcgtg	gaaggtttggtccacggtggggatctc
Y347F	gtgttctttgagaagcgcccgtgcctg	cttctcaaagaacacgccgtcgtcctg
T377S	gacgagtctttcgacaacatcgtggcg	gtcgaaagactcgtccacggtggggat
W76A	ttgaacgctgatcagtaccagtcgatc	ctgatcagcgttcaaggattccagcgg
Q431A	gtgggcgctaaacgcagccatttctcc	gcgtttagcgcccacgatgccgcccag
R477A	tcggcggctccgctgcatgagctcaag	cagcggagccgccgagacgatctcggt
D342A	taccaggctgacggcgtgttctacgag	gccgtcagcctggtaatgatcgaaatt
D343A	caggacgctggcgtgttctacgagaag	cacgccagcgtcctggtaatgatcgaa
Cloning (Wild-type)	actttaagaaggagatatacatatggccaaggcagtggggctgcgtcgc	agtggtggtggtggtggtgctcgaggtagatcttgcgttcgctcgccgg

All primer pairs are represented from 5′ to 3′.

The forward primer for wild-type was designed to eliminate the N-terminal signal sequence predicted by the signalP6.0 server (https://services.healthtech.dtu.dk/services/SignalP-6.0/)[1].

The positions of the mutations are underlined.

Table S5. Crystallographic data collection and refinement statistics of XccOpgD

**Data set**	**XccOpgD-β-1,2-glucan (D379N mutant)**
**Data collection**	
Beamline	KEK NW-12A
Space group	$P6_5$
Unit cell parameters (Å, °)	a = 171.88<br>b = 171.88<br>c = 128.69<br>α, β = 90<br>γ = 120
Resolution (Å)[a]	48.723–2.25 (2.29–2.25)
Total reflections[a]	1064039 (51345)
Unique reflections[a]	102253 (5064)
Completeness (%)[a]	100 (100)
Multiplicity[a]	10.4 (10.1)
Mean $I/\sigma(I)$[a]	11.6 (2.9)
R_{merge} (%)[a]	15.8 (85.6)
R_{pim} (%)[a]	7.6 (41.6)
$CC_{1/2}$[a]	(0.830)
**Refinement**	
Resolution (Å)	48.723–2.25
No. of reflections	102207
No. of atoms	9012
No. of water molecules	541
R_{work}/R_{free} (%)	17.3/17.6
No. of asymmetric units	2
r.m.s.d. from ideal values	
Bond lengths (Å)	0.0089
Bond angles (°)	1.5083
Average B-factors (Å^2)	
Protein (chain A/B)	31.1/34.8
Ligand	
β-1,2-Glucan	39.11
Solvent	32.5
Ramachandran plot (%)	
Favoured	97.0
Allowed	3.0
Outlier	0.0
**PDB entry**	8X18

[a]Values in parentheses represent the highest resolution shell.

Supplementary Notes

Note S1. The abbreviation for SGL

The abbreviation BGL for beta-glucanase is indistinguishable from beta-glucosidase. In addition, there appears to be no standard abbreviation for beta-glucanase. Therefore, as an acronym that would be easy to distinguish linkage positions, "S" representing sophoro (-oligosaccharide), an alternative name for β-1,2-gluco-oligosaccharide was adopted as the abbreviation of β-1,2-glucanase.

Note S2. Reverse reaction of XccOpgD

Whether reverse reaction occurs or not was examined. The reaction was performed in the mixture containing 5% purified product of XccOpgD and 3% β-1,2-glucooligosaccharide mixture produced by hydrolysis of β-1,2-glucans with EcOpgD ($Sop_{6\text{-}10}$) and 1 mg/mL XccOpgD. If the reverse reaction of XccOpgD occurs to some extent, spots corresponding to DPs of larger than around 20 would be observed in a TLC plate after enough reaction time. However, such spot was not observed after 24 h-reaction (data not shown). Therefore, the equilibrium of the reaction of XccOpgD may be highly biased to product side.

Note S3. Molecular mass of products released by XccOpgD

Molecular masses of cyclic-glucans with DP 32 and 48 (C32 and C48, respectively) were detected by ESI-MS (Fig. 1b). They were also detected when the purified CβG16α was analysed by ESI-MS (Extended Data Fig. 1), indicating that the detection of C32 and C48 is derived from detecting two or three CβG16αs with one or two water molecules. This observation is consistent with the NMR results and Michaelis complex of XccOpgD. In addition, a weak peak likely representing C17 was detected. However, this peak was too weak to confirm the presence of C17 (Fig. 1b). Furthermore, HSQC data demonstrated clearly that the purified product is hexadecaose. Overall, the product of XccOpgD is α-1,6-cyclized β-1,2-hexadecaose.

Note S4. The importance of plus subsites for the reaction of XccOpgD.

Subsites +1 to probably +6 are vital for the reaction of XccOpgD. This feature was implied at our X-ray crystallographic data (data not shown). As described in Methods section, high concentration (20%) of linear β-1,2-glucans (average DP17.7 and Mw/Mn < 1.2) was used for soaking the crystals of XccOpgD. We also obtained the structure of XccOpgD using 5% and 10% linear β-1,2-glucans for soaking. However, only weak electron densities are observed at subsites −8 to around −13, suggesting that the substrates preferentially bind to subsites +6 to −7. This result also suggests some plus subsites are vital for the reaction (substrate binding) and that DP16–17 are insufficient as substrates in lengths. Necessity of the high concentration of the substrate for soaking is probably because the ratio of DP22 is very low in the substrates considering the low Mw/Mn value.

Note S5. Occupancy of 6-hydroxy group of the Glc moiety at subsite –16

The 6-OH group of the Glc moiety at subsite –16 seems to be able to move to the appropriate position for the reaction. The rotamer of the 6-OH in the co-crystal in this study is *gt* conformation, while the rotamer for the reaction is *gg* conformation. According to MD simulation by Abe et al[2], both rotamers in 6-OH groups of Sop_ns exist comparably both in free and in complex structures.

Note S6. Differences in reaction mechanisms between GH enzymes and other enzymes (glycosyltransferases, glycoside phosphorylases and glycosynthases)

Structural, and functional analyses of XccOpgD revealed an anomer-inverting transglycosylation mechanism (Figure 1c). Among glycoside hydrolases, reactions are categorized into two canonical types, anomer-retaining GH and anomer-inverting GH (Figure 1). In both types, hydrolysis occurs when a nucleophile in the last step of the reaction is water (Figures 1a, b). In contrast, transglycosylation has been found only in an anomer-retaining mechanism[3] (Figure 1a). Therefore, the XccOpgD reaction (Figure 1c) is the first-discovered anomer-inverting transglycosylation.

Transglycosylation is a reaction catalyzing inter- or intra-molecular substitution of the anomeric position of glycosides[3]. Thus, the anomer-inverting transglycosylation should be clearly distinguished from the other anomer-inverting glycosyl transfer, namely forward reactions of glycosyltransferases[4–6], reverse reactions of glycoside phosphorylases[7] and artificially engineered glycosynthase[8]. These reactions depend on thermodynamical advantage (ability of leaving groups) of doner substrates such as sugar-mono or diphosphonucleotide, sugar 1-phosphate and sugar 1-fluoride. This is related to the fact that they have only one catalytic residue. In contrast, another catalytic residue is needed to make a nucleophile by attracting a proton from a hydroxy group of a glycoside (a poor leaving group) in an acceptor for transglycosylases.

Experimental Methods

Cloning and purification of XccOpgD

The gene encoding XccOpgD (GenBank: AAM43366.1) was amplified by PCR with the primer pair shown in Table S4 using PrimeSTAR Max (Takara Bio) and a genomic DNA of *X. campestris* pv. *campestris* (DSM3568, the Leibniz Institute, Germany) as the template. The forward primer was designed to eliminate the N-terminal signal sequence predicted by the SignalP6.0 server (https://services.healthtech.dtu.dk/services/SignalP-6.0/)[1]. The amplified gene was inserted between the XhoI and NdeI sites of the pET30a vector by the SLiCE method[9] to add a C-terminal His_6-tag to the target protein. The constructed plasmid was transformed into *E. coli* Rosseta2 (DE3) cells and the transformants were cultured in 1 L Luria-Bertani medium containing 30 mg/L kanamycin at 37 °C until the absorbance at 600 nm reached 0.6. Expression was induced by adding isopropyl β-d-1-thiogalactopyranoside to a final concentration of 0.1 mM, and cells were cultured at 20 °C for 24 h. The cells were centrifuged at 7000 *g* for 10 min and resuspended in 50 mM Tris-HCl buffer (pH 7.5). The resuspended cells were disrupted by sonication, and the sample was centrifuged at 33000 *g* for 15 min to obtain a cell extract. The cell extract was loaded onto a HisTrap™ FF crude column (5 mL; Cytiva) pre-equilibrated with buffer (50 mM Tris-HCl, 500 mM NaCl and 20 mM imidazole, pH 7.5). After washing the column with the same buffer, the target protein was eluted with a linear gradient of 20–300 mM imidazole in a buffer containing 50 mM Tris-HCl (pH 7.5) and 500 mM NaCl. Amicon Ultra 30,000 molecular weight cutoff centrifugal filters (Merck, NJ, USA) were used to concentrate a portion of the fractionated protein and exchange the buffer to 50 mM Tris-HCl (pH 7.5) containing 50 mM NaCl. Each purified protein migrated as a single band of 60 kDa on SDS-PAGE gels, which is consistent with the theoretical molecular mass of XccOpgD (57851.479 Da). Concentrations of purified enzymes were calculated from the absorbance at 280 nm[10]. The extinction coefficient of XccOpgD at 280 nm is 115270 $mol^{-1}cm^{-1}$.

β-1,2-Glucans used for experiments

Linear β-1,2-glucans with an average DP of 121 calculated from their number average molecular weight (Mn) were used for TLC analysis, product preparation for ESI-MS and NMR, investigation of general properties and kinetic analysis. The average DP of linear β-1,2-glucans used for crystallization to obtain the Michaelis complex of XccOpgD was 17.7 based on the Mn. All linear β-1,2-glucans used in this study were prepared according to several references[11,12].

TLC analysis

XccOpgD (0.55 mg/mL) was incubated with 1% linear β-1,2-glucan in 2.5 mM Tris-HCl buffer (pH 7.5) at 37 °C for 24 h (sample 1). One microliter 1 mg/mL BtBGL was added to 10 μL sample 1 and incubated at 37 °C for 24 h (sample 2). One microliter 1 mg/mL BtBGL and 1 μL 3 mg/mL CpSGL

were added to sample 1 and incubated at 37 °C for 24 h (sample 3). The reaction mixtures (1 μL) were spotted onto TLC Silica Gel 60 F_{254} (Merck) plates. The plates were developed with 72% acetonitrile once. The plates were then soaked in a 5% (w/v) sulfuric acid/methanol solution and heated in an oven until the spots were clearly visualized. A mixture of β-1,2-glucooligosaccharides was prepared as a marker by incubating β-1,2-glucooligosaccharides with DPs 3–7 and linear β-1,2-glucan in 1 mM sodium phosphate containing 1,2-β-oligoglucan phosphorylase from *Listeria innocua*[13].

ESI-MS

XccOpgD (0.135 mg/mL) was incubated with 1% linear β-1,2-glucan in 5 mM Tris-HCl buffer (pH 7.5) at 37 °C for 24 h (sample 1). One microliter 10 mg/mL BtBGL was added to 49 μL sample 1 and incubated at 37 °C for 24 h (sample 2). 0.24 mg/mL BtBGL and 0.060 mg/mL CpSGL were incubated with 1% purified CβG16α at 37 °C for 24 h (sample 3). Amberlite MB4 (Organo) was added to samples 1 and 2 to remove ionic compounds. The resultant solutions were diluted 100-fold with a solvent (methanol/water = 1/1, v/v) containing 5 mM ammonium acetate. After filtration, the samples were loaded onto the Sciex X500 R QTOF (Sciex) in the positive mode at a 20 μL/min flow rate.

NMR

The enzymatic reaction of 20 μg/mL XccOpgD, 1% linear β-1,2-glucan and 4 mM Tris-HCl buffer (pH 7.5) was incubated at 30°C overnight. Then, 10 μL 109 mg/mL BtBGL and 250 μL 1M bis-Tris buffer (pH 5.5) was added and incubated overnight. The reaction product was purified by size-exclusion chromatography using a Toyopearl HW-40F column (~2 L gel). The sample was eluted with distilled water after the injection of the reaction mixture (~10 mL). The eluates were fractionated into 30-mL portions, and the fraction containing only the main product of the reaction by XccOpgD was lyophilized. One- (^{1}H and ^{13}C) and two-dimensional (double-quantum-filtered COSY, heteronuclear single-quantum coherence (HSQC), heteronuclear multiple-bond correlation (HMBC) and HSQC-TOCSY) NMR spectra of the products were acquired in D_2O at 298 K using a Bruker Avance 800 MHz spectrometer (Bruker, MA, USA) with *t*BuOH (δ 1.23 ppm for ^{1}H, and δ 31.30 ppm for ^{13}C) as an internal standard. The H-1 chemical shift at the α-anomer was assigned by referring to the Karplus curve[14]. The H-2 chemical shift was assigned based on double-quantum-filtered COSY spectra. The C-1 and C-2 chemical shifts were assigned using HSQC spectra and based on the assignment of H-1 and H-2 proton signals. The H-6 and C-6 chemical shifts were determined using DEPT135, HSQC and HSQC-TOCSY data. Linkage positions between glucose units were determined by detecting cross-peaks in the HMBC spectrum.

General properties

The optimum pH of XccOpgD (0.1 mg/mL) activity was determined by incubating the protein in various 20 mM buffers (sodium acetate-HCl, pH 4.0–5.5; bis-Tris-HCl, pH 5.5–7.5; Tris-HCl, pH 7.5–9.0; glycine, pH 9.0–10.0) containing 0.25% linear β-1,2-glucan (average DP121) at 30 °C for 10 min and then heating at 100 °C for 5 min to terminate the reaction. It was confirmed that both Tris-HCl and bis-Tris-HCl did not affect the transglycosylation activity according to comparison with sodium phosphate. BtBGL and bis-Tris-HCl (pH 5.5) were added (final concentrations were 1 mg/mL and 83 mM, respectively) to convert non-cyclized β-1,2-glucan into glucose. The amounts of produced glucose were measured by the GOPOD method[15] to determine the amounts of residual linear β-1,2-glucan. The conversion rates were calculated as subtracts between weights of the initial and residual substrates in the reaction by XccOpgD. The optimum temperature was determined by performing the reactions in 20 mM Tris-HCl buffer (pH 7.5) at each temperature (0–70 °C) for 10 min and then heated at 100 °C for 5 min to terminate the reaction. The conversion rates were calculated using the same approach to determine the optimum pH.

Substrate specificity

XccOpgD (0.61 mg/mL) was incubated in 5 mM Tris-HCl buffer (pH 7.5) containing each substrate (1% glucomannan, Neogen, MI, USA; 1% polygalacturonic acid, Neogen; 1% carboxymethyl cellulose, Merck; 1% soluble starch, FUJIFILM Wako Chemical Corporation, Osaka, Japan; 0.5% carboxymethyl curdlan, Neogen; 0.5% laminarin, Merck; 0.5% lichenan, Neogen; 1% arabinogalactan, Neogen; 0.5% barley β-glucan, Neogen; 1% tamarind-xyloglucan, Neogen; or 1% arabinan, Neogen) at 30 °C for 24 h. The reaction patterns were analysed by TLC.

Kinetic analysis

The kinetic parameters for linear β-1,2-glucans were determined by performing the enzymatic reaction in a 20 μL reaction mixture containing 0.2 mg/mL XccOpgD, 0.007–0.2 mM linear β-1,2-glucan and 20 mM Tris-HCl (pH 7.5) at 30 °C for 10 min. The reaction was stopped by heat treatment at 100 °C for 5 min. BtBGL and bis-Tris-HCl (pH 5.5) were added (final concentrations 1 mg/mL and 16.7 mM, respectively) to convert non-cyclized β-1,2-glucans into glucose. The reaction products were reduced using a one-fifth volume of 1 M $NaBH_4$. The same volume of 1 M acetate as that of the 1M $NaBH_4$ solution was added to each sample to neutralize $NaBH_4$-treated solutions. The samples were then treated with 0.6 mg/mL of BtBGL and 0.15 mg/mL CpSGL at 30 °C for 24 h to convert CβG16α into 11 glucoses and a non-degradable glucopentaose. The main residual oligosaccharide was identified to be glucopentaose by ESI-MS.

Colour development of the reaction mixtures was performed using the GOPOD method[15] to quantify the concentration of glucose derived from the products released by XccOpgD. Molar concentrations of linear β-1,2-glucan substrates were calculated based on the Mn of the substrates. Kinetic parameters

of XccOpgD were determined by fitting experimental data to the Michaelis-Menten equation, $v/[E]_0 = k_{cat} [S]/(K_m + [S])$, where v is the initial velocity, $[E]_0$ is the enzyme concentration, [S] is the substrate concentration, k_{cat} is the turnover number, and K_m is the Michaelis constant. Each analysis was performed in triplicate, and the medians were used for regression.

Crystallography

As described above, XccOpgD (D379N) was purified using a HisTrap™ FF crude column. The crystal of the D379N mutant for the linear β-1,2-glucan complex was obtained at 20 °C after a month by mixing 1 μL D379N mutant (7.0 mg/mL) with 1 μL reservoir solution comprising 0.1 M bis-Tris buffer (pH 5.5) and 2 M $(NH_4)_2SO_4$. The complex crystal of D379N was soaked in the reservoir solution supplemented with 30% (w/v) glycerol and 20% (w/v) linear β-1,2-glucan. The crystal was kept at 100 K in a nitrogen-gas stream during data collection. The X-ray diffraction data was collected on a beamline (BL-5A) at Photon Factory (Tsukuba, Japan). The diffraction data of crystal of the linear β-1,2-glucan-bound XccOpgD (D379N) was collected at 1.0 Å and processed with X-ray Detector Software (http://xds.mpimf-heidelberg.mpg.de/)[16] and the Aimless program (http://www.ccp4.ac.uk/). The initial phase of XccOpgD structure was determined by molecular replacement using the Alphafold2 predicted XccOpgD as a model structure. Molecular replacement, auto model building and refinement were performed using MOLREP, Buccaneer, REFMAC5 and Coot programs, respectively (http://www.ccp4.ac.uk/)[17–20]. Crystallographic data collection and refinement statistics are summarized in Table S5. All visual representations of the structures were prepared using PyMOL (https://pymol.org/2/).

Mutational analysis

The plasmids for producing XccOpgD mutants were constructed using a PrimeSTAR mutagenesis basal kit (Takara Bio) according to the manufacturer's instructions. PCRs were performed using appropriate primer pairs (Table S4) and the XccOpgD plasmid as the template. Transformation into *E. coli* Rosseta2 (DE3) and the expression and purification of XccOpgD mutants were performed using the same methods described for wild-type XccOpgD preparation. The enzymatic reactions of XccOpgD mutants were performed similarly to determine the specific activity at 0.1 mM substrate concentration. The final assay concentration of the mutants and reaction times were 0.023–2.6 mg/mL and 0–6.5 h, respectively, depending on the mutants. Colour development was performed in the same way as described in Kinetic analysis.

CD spectra

CD spectra were recorded between 200–250 nm using a J820 spectropolarimeter (JASCO). Each sample contained 2 mM Tris-HCl (pH 7.5) and a mutant (0.011 mg/mL).

Sequence analysis

All sequence alignment was performed using Clustal Omega[21] and visualized using the ESPript 3.0 server (http://espript.ibcp.fr/ESPript/ESPript/)[22].

Supporting References

(1) Teufel, F.; Almagro Armenteros, J. J.; Johansen, A. R.; Gíslason, M. H.; Pihl, S. I.; Tsirigos, K. D.; Winther, O.; Brunak, S.; von Heijne, G.; Nielsen, H. SignalP 6.0 predicts all five types of signal peptides using protein language models. *Nat Biotechnol* **2022**, *40* (July), 1023–1025. https://doi.org/10.1038/s41587-021-01156-3.

(2) Abe, K.; Sunagawa, N.; Terada, T.; Takahashi, Y.; Arakawa, T.; Igarashi, K.; Samejima, M.; Nakai, H.; Taguchi, H.; Nakajima, M.; Fushinobu, S. Structural and thermodynamic insights into β-1,2-glucooligosaccharide capture by a solute-binding protein in *Listeria innocua*. *Journal of Biological Chemistry* **2018**, *293* (23), 8812–8828. https://doi.org/10.1074/jbc.RA117.001536.

(3) Bissaro, B.; Monsan, P.; Fauré, R.; O'Donohue, M. J. Glycosynthesis in a waterworld: new insight into the molecular basis of transglycosylation in retaining glycoside hydrolases. *Biochemical Journal* **2015**, *467* (1), 17–35. https://doi.org/10.1042/BJ20141412.

(4) Lairson, L. L.; Henrissat, B.; Davies, G. J.; Withers, S. G. Glycosyltransferases: structures, functions, and mechanisms. *Annu Rev Biochem* **2008**, *77* (1), 521–555. https://doi.org/10.1146/annurev.biochem.76.061005.092322.

(5) Coutinho, P. M.; Deleury, E.; Davies, G. J.; Henrissat, B. An evolving hierarchical family classification for glycosyltransferases. *J Mol Biol* **2003**, *328* (2), 307–317. https://doi.org/10.1016/S0022-2836(03)00307-3.

(6) Campbell, J. A.; Davies, G. J.; Bulone, V.; Henrissat, B. A classification of nucleotide-diphospho-sugar glycosyltransferases based on amino acid sequence similarities. *Biochemical Journal* **1997**, *326* (3), 929–939. https://doi.org/10.1042/bj3260929u.

(7) Nakai, H.; Kitaoka, M.; Svensson, B.; Ohtsubo, K. Recent development of phosphorylases possessing large potential for oligosaccharide synthesis. *Curr Opin Chem Biol* **2013**, *17* (2), 301–309. https://doi.org/10.1016/j.cbpa.2013.01.006.

(8) Honda, Y.; Kitaoka, M. The First Glycosynthase derived from an inverting glycoside hydrolase. *Journal of Biological Chemistry* **2006**, *281* (3), 1426–1431. https://doi.org/10.1074/jbc.M511202200.

(9) Motohashi, K. A simple and efficient seamless DNA cloning method using SLiCE from *Escherichia coli* laboratory strains and its application to SLiP site-directed mutagenesis. *BMC Biotechnol* **2015**, *15* (1), 47. https://doi.org/10.1186/s12896-015-0162-8.

(10) Pace, C. N.; Vajdos, F.; Fee, L.; Grimsley, G.; Gray, T. How to measure and predict the molar absorption coefficient of a protein. *Protein Sci* **1995**, *4*, 2411–2423. https://doi.org/10.1002/pro.5560041120.

(11) Kobayashi, K.; Nakajima, M.; Aramasa, H.; Kimura, S.; Iwata, T.; Nakai, H.; Taguchi, H. Large-scale preparation of β-1,2-glucan using quite a small amount of sophorose. *Biosci Biotechnol Biochem* **2019**, *83* (10), 1867–1874. https://doi.org/10.1080/09168451.2019.1630257.

(12) Nakajima, M.; Tanaka, N.; Kobayashi, K.; Nakai, H.; Kimura, S.; Iwata, T.; Taguchi, H. Enzymatic control and evaluation of degrees of polymerization of β-(1→2)-glucans. *Anal Biochem* **2021**, *632*, 114366. https://doi.org/10.1016/j.ab.2021.114366.

(13) Nakajima, M.; Toyoizumi, H.; Abe, K.; Nakai, H.; Taguchi, H.; Kitaoka, M. 1,2-β-oligoglucan phosphorylase from *Listeria innocua*. *PLoS One* **2014**, *9* (3), e92353–e92353. https://doi.org/10.1371/journal.pone.0092353.

(14) Minch, M. J. Orientational dependence of vicinal proton□proton NMR coupling constants: The Karplus relationship. *Concepts Magn Reson* **1994**, *6* (1), 41–56. https://doi.org/10.1002/cmr.1820060104.

(15) Kobayashi, K.; Shimizu, H.; Tanaka, N.; Kuramochi, K.; Nakai, H.; Nakajima, M.; Taguchi, H. Characterization and structural analyses of a novel glycosyltransferase acting on the β-1,2-glucosidic linkages. *J Biol Chem* **2022**, *298* (3), 101606. https://doi.org/10.1016/j.jbc.2022.101606.

(16) Kabsch, W. XDS. *Acta Crystallogr D Biol Crystallogr* **2010**, *D66* (2), 125–132. https://doi.org/10.1107/S0907444909047337.

(17) Vagin, A.; Teplyakov, A. Molecular replacement with *MOLREP*. *Acta Crystallogr D Biol Crystallogr* **2010**, *66* (1), 22–25. https://doi.org/10.1107/S0907444909042589.

(18) Cowtan, K. The *Buccaneer* software for automated model building. 1. Tracing protein chains. *Acta Crystallogr D Biol Crystallogr* **2006**, *62* (9), 1002–1011. https://doi.org/10.1107/S0907444906022116.

(19) Murshudov, G. N.; Vagin, A. A.; Dodson, E. J. Refinement of macromolecular structures by the maximum-likelihood method. *Acta Crystallogr D Biol Crystallogr* **1997**, *53* (3), 240–255. https://doi.org/10.1107/S0907444996012255.

(20) Emsley, P.; Cowtan, K. *Coot*: model-building tools for molecular graphics. *Acta Crystallogr D Biol Crystallogr* **2004**, *60* (12), 2126–2132. https://doi.org/10.1107/S0907444904019158.

(21) Sievers, F.; Higgins, D. G. Clustal Omega for making accurate alignments of many protein sequences. *Protein Science* **2018**, *27* (1), 135–145. https://doi.org/10.1002/pro.3290.

(22) Robert, X.; Gouet, P. Deciphering key features in protein structures with the new ENDscript server. *Nucleic Acids Res* **2014**, *42* (W1), W320–W324. https://doi.org/10.1093/nar/gku316.
